# Plectin plays a role in the migration and volume regulation of astrocytes: a potential biomarker of glioblastoma

**DOI:** 10.1186/s12929-024-01002-z

**Published:** 2024-01-23

**Authors:** Maja Žugec, Borut Furlani, Maria J. Castañon, Boštjan Rituper, Irmgard Fischer, Giuseppe Broggi, Rosario Caltabiano, Giuseppe M. V. Barbagallo, Michelino Di Rosa, Daniele Tibullo, Rosalba Parenti, Nunzio Vicario, Saša Simčič, Victorio Martin Pozo Devoto, Gorazd B. Stokin, Gerhard Wiche, Jernej Jorgačevski, Robert Zorec, Maja Potokar

**Affiliations:** 1 Laboratory of Neuroendocrinology-Molecular Cell Physiology, Institute of Pathophysiology, Faculty of Medicine, University of Ljubljana, Ljubljana, Slovenia; 2 Max Perutz Laboratories, Department of Biochemistry and Cell Biology, University of Vienna, Vienna, Austria; 3 Department of Medical and Surgical Sciences and Advanced Technologies “G.F. Ingrassia”, University of Catania, Catania, Italy; 4 Department of Biomedical and Biotechnological Sciences, University of Catania, Catania, Italy; 5 Institute of Microbiology and Immunology, Faculty of Medicine, University of Ljubljana, Ljubljana, Slovenia; 6 International Clinical Research Center (ICRC), St. Anne’s University Hospital in Brno, 625 00 Brno, Czech Republic; 7 Department of Histology and Embryology, Faculty of Medicine, Masaryk University, Brno, Czech Republic; 8 Institute for Molecular and Translational Medicine, Faculty of Medicine and Dentistry, Palacky University Olomouc, Olomouc, Czech Republic; 9 Department of Neurology, Gloucestershire Royal Hospital, Gloucestershire NHS Foundation Trust, Gloucester, UK; 10 Celica Biomedical, Ljubljana, Slovenia

**Keywords:** Astrocyte, Glioblastoma, Plectin, Aquaporin 4, Intermediate filaments, Cytoskeleton, Edema, Cell volume, Cell migration

## Abstract

**Background:**

The expression of aquaporin 4 (AQP4) and intermediate filament (IF) proteins is altered in malignant glioblastoma (GBM), yet the expression of the major IF-based cytolinker, plectin (PLEC), and its contribution to GBM migration and invasiveness, are unknown. Here, we assessed the contribution of plectin in affecting the distribution of plasmalemmal AQP4 aggregates, migratory properties, and regulation of cell volume in astrocytes.

**Methods:**

In human GBM, the expression of glial fibrillary acidic protein (*GFAP)*, *AQP4* and *PLEC* transcripts was analyzed using publicly available datasets, and the colocalization of PLEC with AQP4 and with GFAP was determined by immunohistochemistry. We performed experiments on wild-type and plectin-deficient primary and immortalized mouse astrocytes, human astrocytes and permanent cell lines (U-251 MG and T98G) derived from a human malignant GBM. The expression of plectin isoforms in mouse astrocytes was assessed by quantitative real-time PCR. Transfection, immunolabeling and confocal microscopy were used to assess plectin-induced alterations in the distribution of the cytoskeleton, the influence of plectin and its isoforms on the abundance and size of plasmalemmal AQP4 aggregates, and the presence of plectin at the plasma membrane. The release of plectin from cells was measured by ELISA. The migration and dynamics of cell volume regulation of immortalized astrocytes were assessed by the wound-healing assay and calcein labeling, respectively.

**Results:**

A positive correlation was found between plectin and AQP4 at the level of gene expression and protein localization in tumorous brain samples. Deficiency of plectin led to a decrease in the abundance and size of plasmalemmal AQP4 aggregates and altered distribution and bundling of the cytoskeleton. Astrocytes predominantly expressed P1c, P1e, and P1g plectin isoforms. The predominant plectin isoform associated with plasmalemmal AQP4 aggregates was P1c, which also affected the mobility of astrocytes most prominently. In the absence of plectin, the collective migration of astrocytes was impaired and the dynamics of cytoplasmic volume changes in peripheral cell regions decreased. Plectin’s abundance on the plasma membrane surface and its release from cells were increased in the GBM cell lines.

**Conclusions:**

Plectin affects cellular properties that contribute to the pathology of GBM. The observed increase in both cell surface and released plectin levels represents a potential biomarker and therapeutic target in the diagnostics and treatment of GBMs.

**Supplementary Information:**

The online version contains supplementary material available at 10.1186/s12929-024-01002-z.

## Background

Glioblastoma (GBM), also known as grade IV glioma, is the most aggressive and lethal form of diffuse glioma with a global incidence of 1/33,000 people (i.e. ∼ 60% of all brain tumors in adults) and a predicted medium length of survival of 12–15 months [1, 2]. GBMs arise from neural stem cells (NSCs), NSC-derived astrocytes and oligodendrocyte precursor cells in the subventricular zone adjacent to the lateral ventricles, and in the subcortical white matter [1]. The spread of GBM cells to the surrounding brain tissue proceeds along the blood vessels and axons [3]; however, the details regarding their strategies to spread throughout the central nervous system (CNS) are vaguely understood [3].

During malignant transformation, an astrocyte is reprogrammed into an oncogenic phenotype through extensive changes in the expression and distribution of a number of proteins, including those forming intermediate filaments (IFs), in particular glial fibrillary acidic protein (GFAP) and its isoforms [4]. Moreover, increased expression of aquaporin 4 (AQP4) and decreased expression of tumor suppressor p53 and α/β-tubulin have been detected in GBM [3, 5, 6]. The impact of IFs on various aspects of GBM biology is unclear, because individual subcellular topography and expression levels of IFs vary considerably among GBM samples [6]. By associating with different types of IFs, interconnecting them with the plasma membrane and cellular organelles, and mediating their interactions with actin filaments (AFs) and microtubules (MTs [7, 8], the cytolinker protein plectin is one of the key regulators of cytoskeleton plasticity including IF networking. However, plectin’s role in astrocytes and GBM, remains unexplored. Plectin is encoded by a single gene (PLEC) which includes several variable first coding exons [9]. Alternative splicing of the gene creates the basic protein isoforms P1, P1a, P1b, P1c, P1d, P1e, P1f, P1g, P1h,i,j, and P1k, which are expressed in variable proportions in most mammalian tissues and cell types [7, 9, 10]. The distinct N-terminal head domains of these isoforms determine their differential subcellular targeting [7]. P1c was identified as the most abundantly expressed isoform in brain followed by P1e and P1g [10]. Plectin is expressed in the majority of astrocytes and prominently in all ependymal cells, including the choroidal epithelial cells, tanycytes, and endothelial cells, suggesting a role of plectin in blood–brain barrier function [8, 11, 12]. Plectin is also a scaffolding platform for signaling [7], leading us to propose that it plays important roles in a number of astrocyte processes, including cell migration, ion and water homeostasis, and modulation of synaptic plasticity [8]. Similarly, alterations in the expression and localization of plectin may play a decisive role in the pathophysiology and progression of GBM and in brain edema dynamics. The blood–brain barrier turns leaky in brain tumors, allowing the influx of water, which then exits the brain through the AQP4-rich glia limitans lining the brain ventricles and the brain surface [13]. Besides interlinking IFs and mediating their interaction with MTs and AFs, plectin is important for cytoplasmic positioning of various organelles and proteins [7, 14]. We hypothesize that the positioning of the AQP4 plasma membrane assemblies, which have been proposed to affect migration and invasion of GBM cells [15, 16], may also be mediated by plectin. Similar to plectin, AQP4, the most prevalent aquaporin channel in the CNS, is predominantly expressed in astrocytes, especially in astrocyte endfeet that are in contact with cerebrospinal fluid, as well as in the subarachnoid space and ventricles and at the blood–brain interface [13]. Proteins contributing to the anchoring of AQP4 in the plasmalemma form the dystroglycan (DG) complex, to which plectin has been shown to bind in Schwann cells [17] and myofibers [18]. GBMs are often associated with brain edema, yet the role of AQP4 in astrocytes in the development of edema remains unclear [19]. For this reason, in this study we assessed whether plectin contributes to the regulation of plasmalemmal AQP4 distribution and cytoplasmic volume changes in astrocytes.

In certain tumors, the expression of plectin and/or its cellular distribution is upregulated, e.g., pancreatic ductal adenocarcinoma, non-small cell lung cancer, high-grade epithelial ovarian cancer, prostate cancer, and head and neck squamous cell carcinoma; in the latter case correlating with a lower survival rate [20–27]. Pro-tumorigenic participation of plectin was demonstrated in the proliferation, migration, and invasion of these cancers. Therefore, one aim of our study was to address the important question whether plectin can also be considered a biomarker or a prognostic indicator of GBM.

We report here that the changes in the expression levels of *PLEC* and *AQP4* correlate with each other in human astrocytoma and glioma, and that plectin-AQP4 colocalization is linked to the proliferative index (PI) of GBMs. In addition, we show that plectin regulates the dynamics of plasmalemmal AQP4 aggregates and affects cell migration, apparently also through cell volume regulation in peripheral regions of astrocytes. Further, we identify the major isoforms of plectin expressed in astrocytes and assess their relative proportions. Plectin’s association with plasmalemmal AQP4 aggregates, its increased cell surface localization, and its release from GBM cell cultures, suggest that plectin may serve as a potential biomarker and candidate molecule for the diagnosis and treatment of GBMs.

## Methods

### Human GBM dataset selection and analysis

We searched the NCBI Gene Expression Omnibus (GEO) database (http://www.ncbi.nlm.nih.gov/geo/) to identify transcriptome datasets related to human GBM tumors of different grades. We used the keywords “human,” “glioblastoma,” and “tumor grade” to identify datasets. We accessed the datasets by sample number, as well as by age, sex, and availability of clinical data for the participants. Based on the analysis carried out, we selected GSE108474 [28]. From this dataset, we sorted biopsies from 28 healthy individuals, 148 patients with astrocytoma grade II or grade III, and 221 patients with GBM.

### Human GBM biopsies

Formalin-fixed and paraffin-embedded tissue specimens from patients diagnosed with GBM were obtained from the surgical pathology files at the Anatomic Pathology Section of the University of Catania, Catania, Italy. Diagnosis was made according to the World Health Organization criteria: (1) high-grade glioma with astrocytic morphology; (2) diffuse growth pattern; and (3) foci of necrosis and/or microvascular proliferation. The study and sample collection were conducted according to the guidelines of the Declaration of Helsinki and approved by the Catania 1 Ethics Committee, Catania, Italy (protocol code: 166/2015/PO; 17/12/2015). Four men and four women were included in the study (mean age, 61 years; range, 45–81 years). Samples were divided into two groups according to their PI at diagnosis: (1) high PI (Ki-67 positive cells > 30%) and (2) low PI (Ki-67 positive cells < 30%).

### Cell cultures and tissue slices

Primary human fetal astrocytes cryopreserved at passage one (Innoprot, Derio, Spain), primary mouse astrocytes isolated from cerebral cortices of neonatal (1-day old) *Plec*^*−/−*^ (plectin-null) and wild-type littermates (*Plec*^+*/*+^) obtained from heterozygotes of the mouse strain Plec/29.B6 (N12) [29], and immortalized mouse astrocytes isolated from neonatal *Plec*^*−/−*^*p53*^*−/−*^ and *Plec*^+*/*+^*p53*^*−/−*^ littermates obtained by inter-crossing *Plec*^+*/−*^*p53*^+*/−*^ heterozygotes generated from a previously created mouse line [30], were cultured in high-glucose Dulbecco’s modified Eagle’s medium (Thermo Fisher Scientific, Karlsruhe, Germany) supplemented with 10% fetal bovine serum (FBS), 1 mM sodium pyruvate, 2 mM l-glutamine, 5 U/ml penicillin, and 5 µg/ml streptomycin. Assays performed with immortalized cells were done at low passage (< 10). Immortalized cells (*Plec*^*−/−*^) and (*Plec*^+*/*+^) of different types are a highly valuable tool to study plectin functions as the results obtained using p53 knock-out cell lines are generally identical to the results obtained on cells isolated from plectin knock-out mice [31]. The permanent cell lines U-251 MG and T98G derived from a human malignant GBM were purchased from the European Collection of Authenticated Cell Cultures (ECACC, Public Health England, Salisbury, UK) and maintained in essentially the same growth medium as astrocytes, but with a higher concentration of antibiotics (100 U/ml penicillin, and 100 µg/ml streptomycin). All cell cultures were maintained at 37 °C in a humidified atmosphere of 5% CO_2_ and 95% air. Mouse lines Plec/29.B6 (N12) and *Plec*^+*/−*^*p53*^+*/−*^ were maintained by backcrossing to a C57BL/6 background. Before the experiments, all cell types were tested for astrocyte markers (Additional file 1: Table S1). If not indicated otherwise, cell media and reagents for all the experiments in this study were obtained from Sigma-Aldrich (Merck, Darmstadt, Germany). Cells plated for experiments were used within 3 days after plating.

### Quantitative real-time PCR

Total RNA was isolated from cultured primary and immortalized cortical astrocytes isolated from 1- to 2-day-old *Plec*^+*/*+^, *Plec*^*−/−*^, *Plec*^+*/*+^*p53*^++^and *Plec*^+*/*+^*p53*^*−/−*^ mice. The RNA was purified using Trizol (Invitrogen, Carlsbad, CA, USA), its quality assessed on an agarose bleach gel [32], and the quantity measured using a NanoDrop D-11 + spectrophotometer (DeNovix, Wilmington, DE, USA). cDNA was synthesized from 1 µg of RNA using random hexamer primers (Thermo Fisher Scientific) and SuperScript III (Invitrogen). Real-time polymerase chain reaction (RT-PCR) was performed using SYBR Green II Master Mix in a LightCycler 480 (Roche Diagnostics, Basel, Switzerland). The reactions were run at 95 °C for 2 min followed by 45 cycles of 95 °C for 10 s, 60 °C for 10 s, and 72 °C for 20 s. Data were collected and analyzed using the software supplied with the instrument. The estimated absolute copy numbers of plectin isoforms were calculated from standard curves obtained by serial dilutions of linearized plasmids carrying the target sequences. Data for each exon were from three independent experiments with each data point assayed in duplicate; all samples were normalized to the reference genes hypoxanthine guanine phosphoribosyl transferase 1 (HPRT1) and/or glyceraldehyde-3-phosphate dehydrogenase (GAPDH) and calculations were based on the Pfaffl Method [33]. Primers amplify a region spanning exons 1–2 of cDNA; their sequences are listed in Additional file 1: Table S2.

### Immunohistochemistry of human GBM tissue slices

Immunolabeling was performed by a standard immunohistochemistry protocol [34]. Briefly, samples were warmed to room temperature (RT), deparaffinized in xylene, and hydrated by immersing in a series of graded ethanol. Heat-mediated antigen retrieval was performed in a microwave (10 mM sodium citrate buffer [pH 6.0], 20 min), and blocking solution (10% goat serum and 0.1% Triton X-100 in 1 × phosphate-buffered saline [PBS]) was applied (RT, 1 h) to prevent nonspecific background staining. Primary antibodies were applied overnight at 4 °C and secondary antibodies at RT (1 h). Double immunolabeling was performed sequentially by applying a first pair of primary and secondary antibodies followed by a second pair. Cell nuclei were labeled with DAPI (1 µg/ml; Invitrogen), and slides were embedded in Mowiol 4–88 mounting medium (a mixture of 2.4 g Mowiol 4–88, 6 g glycerol, 6 mL H_2_O and 12 mL 0.2 M Tris–Cl (pH 8.5), heated to 50 °C for 10 min, and then centrifuged at 5000*g* for 15 min; at the end 2.5% DABCO is added to reduce fluorescence fading). The following sera and antibodies (diluted in 0.1% Triton X-100 in 1 × PBS) were used: heat-inactivated (56 °C, 30 min) human serum (acquired from the serum sample bank at the Institute of Microbiology and Immunology, Faculty of Medicine, University of Ljubljana, Ljubljana, Slovenia) and confirmed for the presence of neuromyelitis optica immunoglobulin G antibodies (NMO-IgG, 1:100; Additional file 2: Fig. S1), rabbit antiserum to plectin (#46, 1:100; [30]), rabbit polyclonal antibody to GFAP (1:100; Agilent Technologies, Santa Clara, CA, USA), goat anti-human and goat anti-rabbit IgG conjugated to fluorescent dyes (Alexa Fluor 488 or 546, 1:1000; Invitrogen).

### Immunolabeling of cytoskeleton constituents

Cells plated onto glass coverslips coated with poly-d-lysine (PDL) (1:100) were labeled by a standard immunocytochemistry protocol [35]. To immunolabel vimentin filaments (VFs), cells were fixed and permeabilized in ice-cold 100% methanol (− 20 °C, 10 min); for immunolabeling of cytoplasmic plectin, cells were fixed in 4% formaldehyde (RT, 15 min) followed by permeabilization with 0.1 Triton X-100 (RT, 10 min). Then, standard immunolabeling steps were performed by incubation in blocking solution (3% bovine serum albumin [BSA] and 10% goat serum in 1 × PBS, 1 h, 37 °C) and subsequent labeling with rabbit antiserum to vimentin (1:200; Abcam, Cambridge, UK) and rabbit antiserum to plectin (#46, 1:100), both overnight at 4 °C, followed by secondary antibodies (goat anti-rabbit Alexa Fluor 488 and Alexa Fluor 546, 1:600; Invitrogen) for 45 min at 37 °C. All antibodies were diluted in 3% BSA in 1 × PBS. AFs were labeled in cells fixed with 4% formaldehyde (RT, 15 min) and incubated with 1 × Phalloidin-iFluor 594 (Abcam) diluted in 1% BSA (RT, 30 min). After labeling, the coverslips were mounted onto glass slides using Mowiol 4–88 mounting medium.

### Transfection

Cells were transfected with plasmids encoding the following fusion proteins between different plectin isoforms and fluorescent proteins: P1c2α3α-EGFP [36], P1e-EGFP [36], P1g-EGFP [36], and P1c-mCherry. P1c-mCherry was generated by subcloning of P1c cDNA into pGB7 P1c-mCherry [37]. FuGENE 6 transfection reagent (Promega, Mannheim, Germany) was used according to the manufacturer’s instructions.

### Labeling of plasmalemmal AQP4 aggregates and cell surface plectin microdomains

Cells plated onto glass coverslips coated with PDL (1:100) and laminin (1:100) were labeled as described [38, 39]. Briefly, cells were incubated with 3% BSA in 1 × PBS to prevent nonspecific background signal (37 °C, 1 h). Then, to label AQP4 in the plasmalemma, heat-inactivated NMO-IgG patient serum (1:100) was applied (+ 4 °C, 15 min). The AQP4 antibodies in NMO-IgG serum exhibit a high affinity for the extracellular surface of AQP4 [40]. To label surface plectin, rabbit antiserum to plectin (#46, 1:100) was applied (+ 4 °C, 15 min). Next, cells were fixed in 2% formaldehyde (RT, 15 min), incubated with the appropriate secondary antibodies (37 °C, 45 min), goat anti-human IgG (Alexa Fluor 546, 1:600; Invitrogen), donkey anti-human IgG (Abberior Star Green, 1:600; Abberior, Göttingen, Germany), or goat anti-rabbit IgG (Alexa Fluor 488 or Alexa Fluor 546, 1:600; Invitrogen) and mounted onto glass slides using Mowiol 4–88 mounting medium.

### ELISA quantification

Primary human astrocytes and U-251 MG cells were plated in triplicates on 24-well plates, with a seeding density of 4 × 10^4^ cells per well. After overnight incubation in growth medium to ensure adherence, the medium was replaced with plain DMEM and incubated for an additional 24 h to minimize proliferation. Subsequently, the supernatant (DMEM containing released proteins) was collected and stored frozen at − 80 °C. Prior to performing indirect ELISAs, the protein concentration of samples was determined using a BCA protein assay (Thermo Fisher Scientific). Samples, diluted (1:1) in coating buffer (0.1 M Na2CO3/NaHCO3, pH 9.4), were loaded onto 96-well plates (Thermo Fisher Scientific), incubated for 2 h at RT, followed by washing (3×) in PBS/0.05% Tween-20, and incubated with blocking solution (washing solution plus 2% BSA) for 1 h at RT. The plates were then incubated (2 h, RT) with rabbit anti-plectin antiserum #9 [30] (diluted 1:500 in blocking solution). For subsequent incubation with secondary antibodies, goat anti-rabbit HRP (Thermo Fisher Scientific) was used at a dilution of 1:4000. For signal detection, TMB substrate (Thermo Fisher Scientific) was added for 15 min and the reaction stopped with 2 M sulfuric acid. Immediately after, the absorbance was read at 450 nm using a Multiscan GO spectrophotometer (Thermo Fisher Scientific).

### Imaging of fluorescent immunolabeled samples

Imaging of fluorescently labeled samples was performed using LSM 800 confocal microscope with a Plan-Apochromat 63 × /1.4 oil DIC M27 objective (Carl Zeiss, Oberkochen, Germany) using 405 nm, 488 nm, and 561 nm diode laser excitation. Emission spectra were acquired sequentially with 400–505 nm (DAPI), 505–555 nm (EGFP or Alexa 488), and 555–700 nm (Alexa 546 or mCherry) bandpass emission filters. Z-stacks with a 0.5-µm interval were recorded in samples of immunolabeled intracellular plectin, AFs, VFs, AQP4 aggregates, and surface plectin, and z-stacks with a 1-µm interval were recorded in human GBM tissue slices.

### Wound-healing assay

Cells were seeded on 2-well inserts in 35-mm dishes (Ibidi, Gräfelfing, Germany). After reaching confluence (24 h), the insert was removed, and cells were supplemented with growth medium with reduced FBS content (2%) to minimize the effect of cell proliferation in a repopulation of the insert-free area (the gap) [41]. Cells were kept in 5% CO_2_/95% air at 37 °C. Time-lapse recording (37 °C, 4-h intervals for 28 h) was performed with LSM 800 confocal microscope using a Plan-Apochromat 10 × /NA 0.45 DIC objective.

### Recordings of cell volume changes

Cells plated on PDL- and laminin-coated coverslips were loaded with calcein-acetoxymethyl ester dye (Calcein AM; 1 µM, 23 °C, 15 min; Sigma-Aldrich) and exposed to either iso-osmotic (300 mOsm) or hypo-osmotic (200 mOsm) conditions. Iso-osmolar extracellular solution consisted of 130 mM NaCl (Merck, Germany), 5 mM KCl, 2 mM CaCl_2_, 1 mM MgCl_2_, 10 mM D-glucose, and 10 mM HEPES (pH 7.2). A hypo-osmotic solution was obtained by reducing the osmolarity of the iso-osmolar medium from 300 to 200 mOsm by application of a 100 mOsm solution consisting of 30 mM NaCl, 5 mM KCl, 2 mM CaCl_2_, 1 mM MgCl_2_, 10 mM D-glucose, and 10 mM HEPES (pH 7.2). Images were recorded with LSM 800 confocal microscope using an oil-immersion objective (40×/NA 1.3). Calcein was excited using a diode laser (488 nm), and the emission light was filtered with a bandpass emission filter (400–650 nm). Time-series images were recorded for 30 s before and 90 s after hypotonic stimulation with an acquisition rate of 1 frame/s.

### Image analysis

#### Intracellular distribution of the cytoskeleton and plectin

The analysis was performed with Lpx Filter2d plugins [42] using Fiji version 2.3.0/1.53q (NIH, Bethesda, USA) [43]. Briefly, z-stack images of cells were outlined and skeletonized to calculate the angle against the cell long axis (avgTheta), the level of parallelness among filaments (normAvgRad), an index showing the variations in the angle distribution of the cytoskeleton, and bundling of filaments (skewness of intensity distribution, which indicates bundling) [42]. The subplasmalemmal and perinuclear (PN) occupancy (the amount of cytoskeleton per unit area) was assessed in merged Z-stack images and expressed as the percentage of pixels above the threshold versus pixels representing the selected cell area. The subplasmalemmal area represents the area between the cell outline (obtained from a DIC image) and a corresponding (virtual) 20% or 10% smaller outline (by area) for immunolabeled AFs/VFs, and intracellular plectin, respectively. The PN area represents an area within a twofold area of a cell nucleus, including the nuclear region.

#### Collective cell migration and single-cell mobility

Sequential images were analyzed as described [44]. Briefly, the area devoid of cells (the gap) was outlined, and the collective migration rate was determined by plotting the gap area versus time according to the equation:

$$v_{{{\text{migration}}}} { }\left( {{\mu m}/{\text{h}}} \right) = \frac{{\left| {{\text{slope}}} \right|}}{{2 \times { }l}},$$

where the slope is equal to *dGapArea/dt*; the gap area is defined as the width of the gap times the length of the gap (*l*). The time needed for the gap to close to half the original area (50% of the gap) was calculated as:

$$t_{{1/2 {\text{gap}}}} = \frac{{\text{Initial gap area}}}{{2 \times { }\left| {{\text{slope}}} \right|}}.$$

The analysis of the mobility of single cells, positioned within 150–300 µm from the leading edge, was performed in Fiji. The distance between sequential positions of the cell in two sequential images was measured at the center of cell nuclei. The sum of sequential distances in all recorded time intervals represents the total distance of the cell and was used to calculate the average cell speed.

#### Plamalemmal AQP4 aggregates and cell surface plectin

A 3D object counter plugin in Fiji [43, 45] was used to calculate the abundance of plasmalemmal AQP4 aggregates and surface plectin microdomains in all imaged z-stacks of whole cells. The number of plasmalemmal AQP4 aggregates and surface plectin microdomains was normalized to a cell area of 100 µm^2^. Diameters of plasmalemmal AQP4 aggregates and cell surface plectin microdomains were measured as described [38]. Briefly, the in-focus z plane was chosen, and the diameter was determined by measuring the full-width at half maximum of the fluorescence intensity profile in the horizontal and vertical directions of the equatorial plane of particle.

#### Colocalization analysis

The colocalization between AQP4 and plectin in tissue slices was measured in all imaged z-stacks with the JACoP plugin in Fiji [45].

In cells in culture, the overlap between objects representing plasmalemmal AQP4 aggregates and plectin was determined by the colocalization and 3D object counter plugins in Fiji. The percentage of colocalization reflects the percentage of colocalized objects versus all plasmalemmal AQP4 aggregates in the cell.

#### Measurements of cell volume dynamics

Changes in the cell volume can be deducted from an increase in the fluorescence intensity of the cytosolic calcein, because hypo-osmotic shock leads to calcein dequenching due to dilution of intracellular solutes [46]. Changes in the fluorescence intensity were analyzed in Fiji to calculate the average fluorescence intensity in a region of interest. Subsequent analysis and curve fitting of the data were performed using a custom-written script in the Python programming language (Python Software Foundation, https://www.python.org/). Briefly, florescence intensity data were first smoothened using a Savitzky-Golay filter. Then, to compensate for photobleaching, the linear function was fitted to the baseline (first 30 s) and subtracted from the original data. Subsequently, florescence intensity was normalized to the baseline (*F/F*_0_) and expressed as a percentage (*F/F*_0_ × 100%). The dynamics of cell swelling were modeled with a sigmoid function:

$$F/F_{0} \left( t \right) = \frac{{F_{{{\text{max}}}} }}{{1 + e^{{k\left( {t - t_{0} } \right)}} }},$$

where *F*_max_ corresponds to the maximum florescence intensity, and *k* is the maximal swelling rate [evaluated at the midpoint of the swelling phase (*t*_0_)]. The regulatory volume decrease (RVD) was evaluated by determining the half-time (*t*_1/2_) of the fluorescence decrease following the swelling phase.

### Statistical analysis

Statistical analysis was performed in SigmaPlot 11.0. First, a normality test was performed on the data, then statistical significance was evaluated using the nonparametric Mann–Whitney U test. For a multiple factorial analysis of variance, two-way ANOVA on ranks with the Kruskal–Wallis and Holm-Šídák’s or Dunn’s post hoc tests for non-normally distributed data was applied. Results are presented as medians ± standard error. Significance was considered as follows: *P < 0.05, **P < 0.01, ***P ≤ 0.001*.* The number of replicates and the number of cells analyzed are stated in the figure legends.

## Results

### Expression and localization of plectin correlate with those of AQP4 in humans

**Fig. 1:**
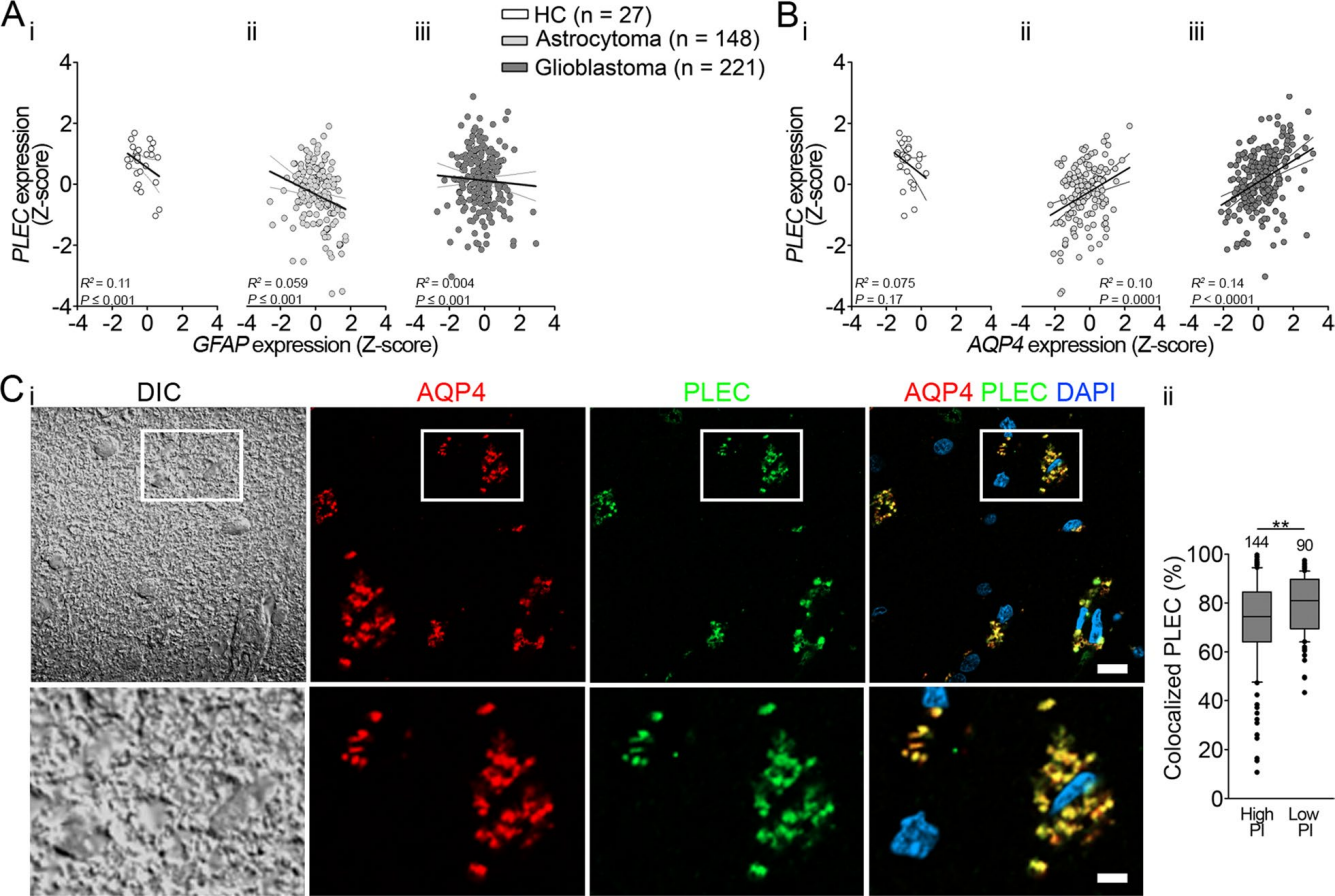
The RNA (Z-score) and the protein localization of aquaporin4 (AQP4) and plectin (PLEC) correlate in human astrocytomas and glioblastomas (GBMs). **A** Gene expression (Z-score) of *PLEC* versus *GFAP* in healthy controls (HC) (A_i_, *y* =  − 0.41 × *x* + 0.55; *R*^2^ = 0.11; *P* ≤ 0.001;), astrocytoma (A_ii_, *y* =  − 0.28 × *x* − 0.31; *R*^2^ = 0.059; *P* ≤ 0.001), and GBM (A_iii_, *y* =  − 0.06 × *x* + 0.13; *R*^2^ = 0.004; *P* ≤ 0.001). Note inverse correlation in all cases. **B** Z-scores of *PLEC* versus *AQP4* in HC. Note, no correlation in HC (B_i_, *y* =  − 0.45 × *x* + 0.33; *R*^2^ = 0.075; *P* = 0.17), and positive correlation in astrocytoma (B_ii_, *y* = 0.34 × *x* − 0.24; *R*^2^ = 0.10; *P* = 0.0001), and GBM (B_iii_, *y* = 0.35 × *x* + 0.07; *R*^2^ = 0.14; *P* < 0.0001). n denotes the number of patients in (**A**) and (**B**). **C**_**i**_ Depiction of immunolabeled AQP4 and PLEC in a slice of human GBM IDH1 wild-type sample with low PI (Ki-67 = 15%). From left to right: DIC image and fluorescence micrographs showing immunolabeled AQP4, immunolabeled PLEC, and an overlay of AQP4, PLEC, and DAPI-labeled nuclei. Yellow color indicates the colocalization signal for AQP4 and PLEC. Sections within the rectangular area are enlarged below. Scale bars: 10 µm (magnified area, 3 µm). **C**_**ii**_ The percentage of colocalized PLEC and AQP4 was lower in GBM with high proliferative index (high PI) in comparison with GBM with low proliferative index (low PI) (**P < 0.01, Mann–Whitney U test). Samples were obtained from patients with GBM IDH1 wild-type (three patients with high PI, two patients with low PI). Numbers above the boxplots represent the number of cells analyzed

GBM RNA transcriptome datasets were used to examine the expression levels of *GFAP*, *AQP4*, and *PLEC* in healthy tissue versus glioma; samples were obtained from 28 healthy human controls (HCs), 148 patients with astrocytoma, and 221 patients with GBM. In particular, we examined whether the RNA expression levels (z-scores) of *PLEC* correlate with levels of *GFAP* and *AQP4*. The results revealed an inverse correlation between *PLEC* and *GFAP* (Fig. 1Ai–iii) in HC, astrocytoma, and GBM samples. Although no correlation was observed between Z-scores of *PLEC* and *AQP4* in HC samples (Fig. 1Bi), the correlation was positive in both astrocytoma and GBM samples (Fig. 1Bii, iii). By inspecting immunohistochemically labeled GBM tissue sections with high and low PI, we detected a high degree of plectin/AQP4 colocalization in both cases (Fig. 1Ci), with a slightly (10%) but significantly higher value observed for low PI compared with high PI GBM (Fig. 1Cii). The colocalization of plectin with AQP4 was higher in comparison with GFAP (Additional file 2: Fig. S2). Previous studies have shown that alterations in the expression of the *AQP4* gene affect the size of supramolecular structures of AQP4, called orthogonal arrays of particles (OAPs), which has subsequently been proposed to contribute to the migratory properties of GBM cells [15, 16, 47]. Our results indicate that plectin might be involved in the positioning of AQP4, which may lead to changes in the size of plasmalemmal AQP4 aggregates.

### The abundance and the size of plasmalemmal AQP4 aggregates of astrocytes depend on plectin expression levels

**Fig. 2:**
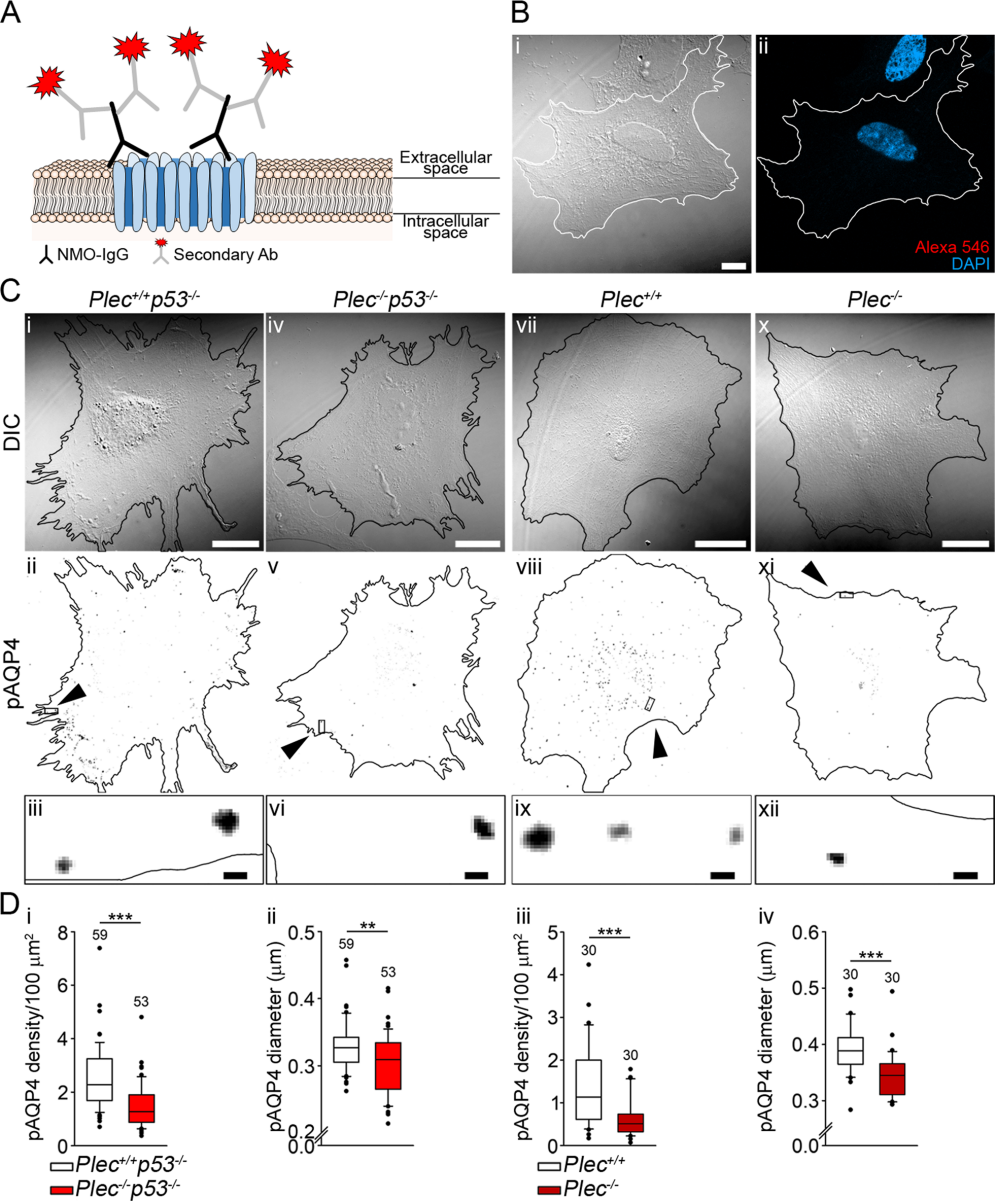
Plectin deficiency reduces the abundance and size of plasmalemmal AQP4 aggregates in immortalized and primary mouse astrocytes. **A** Schematic depiction of a plasmalemmal AQP4 aggregate (pAQP4; cluster of channels in blue) with NMO-IgG serum antibodies (black) bound to the extracellular domain of AQP4, and with secondary antibodies (gray) conjugated Alexa-546 (red) fluorescent dye. **B**_**i**_ and **B**_**ii**_ DIC image of *Plec*^*−/−*^*p53*^*−/−*^ astrocyte and the corresponding fluorescent micrograph with DAPI-labeled cell nuclei. Labeling with secondary (Alexa-546) antibodies alone did not result in measurable fluorescence. **C** DIC images of immortalized (*Plec*^+*/*+^*p53*^*−/−*^ and *Plec*^*−/−*^*p53*^*−/−*^) (**C**_**i**–**vi**_) and primary (*Plec*^+*/*+^ and *Plec*^−/−^) (**C**_**vii**–**xii**_) astrocytes with corresponding inverted fluorescent micrographs depicting labeling of plasmalemmal AQP4 aggregates (black puncta) with enlarged boxed sections (below). Cells are outlined in black. Plasmalemmal AQP4 aggregates in inverted fluorescent micrographs (**C**_**ii**,**v**,**viii**,**xi**_) are displayed as black puncta; arrowheads point to demarcated areas that are enlarged in the panels below (**C**_**iii**,**vi**,**ix**,**xii**_). Scale bars: 20 µm (enlarged areas, 0.5 µm). **D** Quantification of plasmalemmal AQP4 aggregates is revealed in (**C**). Note that immortalized as well as primary astrocytes that express plectin (*Plec*^+*/*+^*p53*^*−/−*^ and *Plec*^+*/*+^, respectively) have more plasmalemmal AQP4 aggregates per 100 µm^2^ (D_i_, D_iii_; ***P ≤ 0.001; Mann–Whitney U test), and their mean size is larger (**D**_**ii**_, **D**_**iv**_; **P < 0.01, ***P ≤ 0.001; Mann–Whitney U test), compared with astrocytes devoid of plectin. The data were obtained from astrocytes isolated from two mice per genotype. Experiments were performed in duplicate. The numbers above the boxplots are the number of cells analyzed

Anchoring of AQP4 in the plasma membrane depends on the extracellular matrix and intracellular plasma membrane associated proteins of the dystrophin–dystroglycan complex which is connected to AFs [48, 49]. Given that plectin regulates the dynamics of AFs [50], we hypothesized that plectin affects the abundance and size of plasmalemmal AQP4 aggregates. We decided to test this hypothesis in immortalized astrocyte cell cultures derived from plectin-deficient (null) and plectin-positive mice that had been crossed into a p53^−/−^ background [51] (see “Methods”). A similar strategy has successfully been used in studies on various other cell types [31, 52]. Labeling of plasmalemmal AQP4 aggregates was achieved using auto-antibodies (NMO-IgG) that specifically bind to AQP4 [40], without producing a non-specific signal (Fig. 2A,B). The analysis revealed that *Plec*^*−/−*^*p53*^*−/−*^ astrocytes exhibited a reduction of plasmalemmal AQP4 aggregates of ∼70% (Fig. 2Ci–vi, D_i_), with plasmalemmal AQP4 aggregates being reduced in diameter by ∼10%, compared with *Plec*^+*/*+^*p53*^*−/−*^ (Fig. 2Ci–iv, D_i_, D_ii_). When the experiment was repeated with primary (*Plec*^+*/*+^) mouse astrocytes, plasmalemmal AQP4 aggregates were found to be more than twice as numerous and 10% larger in *Plec*^+*/*+^ astrocytes compared with plectin-devoid cells (Fig. 2Cvii–xii, D_iii–iv_), confirming the specificity of the observed phenotype. Interestingly, immortalized astrocytes expressed nearly twice as many plasmalemmal AQP4 aggregates than primary mouse astrocytes (*P* < 0.001, Mann–Whitney U test, Fig. 2Di, iii). These results reveal that both the abundance and the size of plasmalemmal AQP4 aggregates depend on the expression of plectin in the cells studied.

**Fig. 3:**
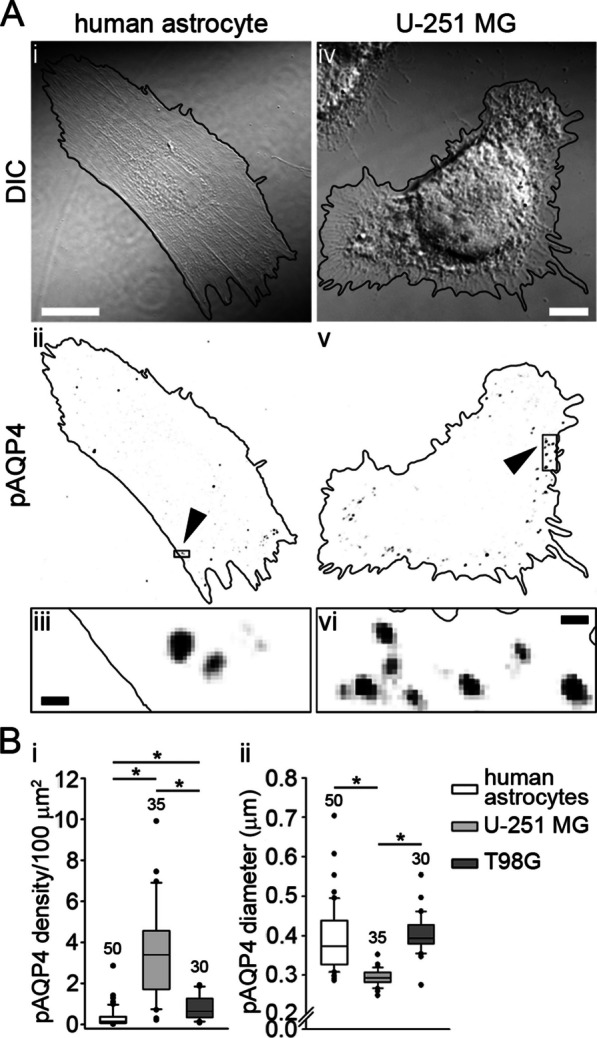
U-251 MG and T98G cells express more plasmalemmal AQP4 aggregates than human astrocytes. **A** DIC images with corresponding inverted fluorescent micrographs and enlarged sections of a human astrocyte (**A**_**i**–**iii**_) and of a U-251 MG cell (**A**_**iv**–**vi**_). Cells are outlined in black, plasmalemmal AQP4 aggregates (pAQP4) are displayed as black dots. Arrowheads in (**A**_**ii**,**v**_) point to boxed areas enlarged in A_iii,vi_. Scale bars: 20 µm (enlarged area, 0.5 µm). **B**_**i**_ Human astrocytes have fewer plasmalemmal AQP4 aggregates in comparison with U-251 MG and T98G cells (*P < 0.05; one-way ANOVA followed by Dunn’s method). **B**_**ii**_ Human astrocytes have larger plasmalemmal AQP4 aggregates in comparison with U-251 MG, but are similar in size to T98G cells (*P < 0.05; one-way ANOVA followed by Dunn’s method). Human astrocytes were isolated from two donors. Experiments were performed in triplicate. The numbers above the boxplots are the number of cells analyzed

When we tested the abundance of plasmalemmal AQP4 aggregates in human astrocytes versus human U-251 MG and T98G GBM cells (using NMO-IgG (Fig. 3A), the results revealed an increase in the density of plasmalemmal AQP4 aggregates by a factor of ∼10 and a reduction in diameter of ∼20% in U-251 MG cells versus human astrocytes (Fig. 3Bi–ii). A higher density of plasmalemmal AQP4 aggregates was observed also in T98G cells (by a factor of ∼2), while their diameter was similar as in human astrocytes (Fig. 3Bi–ii).

### Intracellular distribution of actin and vimentin filaments is affected differently by plectin deficiency

**Fig. 4:**
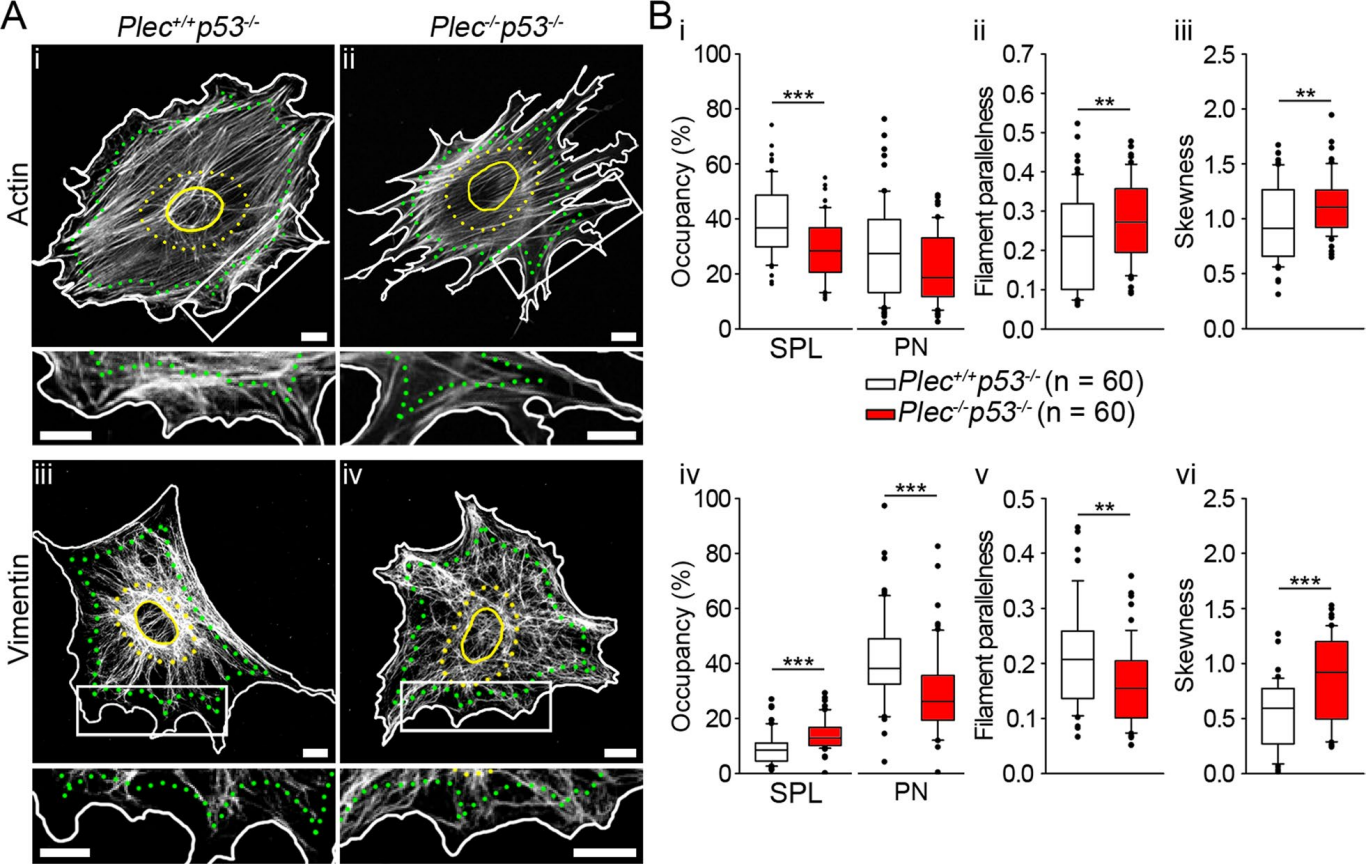
Plectin deficiency affects the distribution of actin filaments (AFs) and vimentin filaments (VFs) in immortalized mouse astrocytes. **A** Immunolabeled AFs (**A**_**i**,**ii**_) and VFs (**A**_**iii**,**iv**_) in *Plec*^+*/*+^*p53*^*−/−*^ (left panels) and *Plec*^*−/−*^*p53*^*−/−*^ (right panels) astrocytes. Boxed areas in whole-cell images are shown enlarged below the corresponding micrographs. Cell outlines are depicted in white, the border of the subplasmalemmal (SPL) area (the area between the cell outline and the line calculated by reducing the cell area by 20%) is demarcated by green puncta. Cell nuclei and perinuclear (PN) region (2 × the size of the nucleus area) are outlined in yellow by a line and puncta, respectively. Scale bars: 10 µm. **B** Statistical analysis of cytoskeletal filament distribution. Note that compared with *Plec*^+*/*+^*p53*^*−/−*^ astrocytes, in *Plec*^*−/−*^*p53*^*−/−*^ astrocytes, AFs are retracted from the SPL area and are more parallel and more bundled throughout the cell cytoplasm (**B**_**i**–**iii**_), whereas VFs are more abundant in the SPL area, less abundant in the PN area, less parallel and more bundled in the cytoplasm (**B**_**iv**–**vi**_). **P < 0.01, ***P ≤ 0.001; Mann–Whitney U test. The data were obtained from astrocytes isolated from two mice per genotype. Experiments were performed in duplicate. n indicates the number of cells analyzed

AQP4 anchoring to dystroglycan complex occurs via an interaction with β-dystroglycan (β-DG) [18, 49]. Given that plectin binds to dystrophin (utrophin) and β-dystroglycan [18], we postulate that plectin may also indirectly affect the positioning of AQP4 in the plasmalemma by mediating crosstalk between VFs, AFs, and MTs. To address this issue, we examined how plectin affects the intracellular distribution of AFs and VFs by immunolabeling AFs and VFs in immortalized astrocytes expressing (*Plec*^+*/*+^*p53*^*−/−*^) or not expressing plectin (*Plec*^*−/−*^*p53*^*−/−*^) (Fig. 4A). The results revealed that in *Plec*^*−/−*^*p53*^*−/−*^ astrocytes, AFs were retracted from the plasma membrane (Fig. 4Bi); their occupancy in the subplasmalemmal area was ∼20% lower compared with *Plec*^+*/*+^*p53*^*−/−*^ cells. In addition, in *Plec*^*−/−*^*p53*^*−/−*^ astrocytes, AFs were arranged more in parallel (Fig. 4Bii) and appeared more bundled (Fig. 4Biii) compared with *Plec*^+*/*+^*p53*^*−/−*^ cells. In contrast to AFs, VFs in *Plec*^*−/−*^*p53*^*−/−*^ astrocytes extended closer to the plasmalemma (the occupancy in the subplasmalemmal was ∼50% higher), redistributed away from the perinuclear region (the occupancy was ∼30% lower), and they were also oriented more irregularly and more bundled in comparison with *Plec*^+*/*+^*p53*^*−/−*^ astrocytes (Fig. 4Biv–vi).

In summary, these results reveal that in astrocytes, plectin is critically involved in the distribution of AFs and VFs, consistent with potential involvement in the positioning of plasmalemmal AQP4 aggregates.

### Among several plectin isoforms expressed in astrocytes, P1c is the most abundant and the one prominently associating with plasmalemmal AQP4 aggregates

**Fig. 5:**
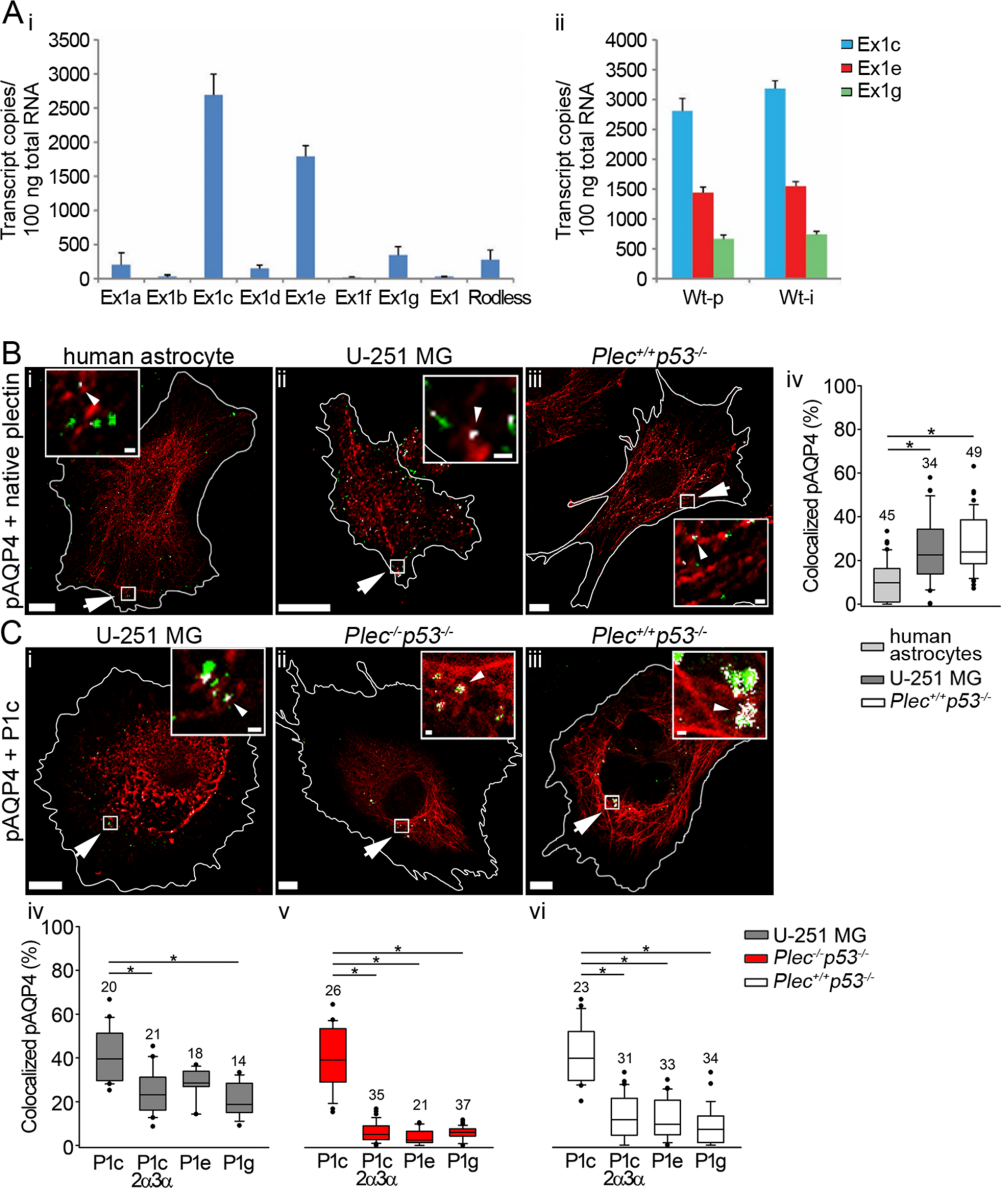
Quantitation of plectin isoform transcripts, plectin-AQP4 aggregates in the plasmalemma colocalization, and preferential targeting of isoform P1c to plasmalemmal AQP4 aggregates*.* **A** Total RNAs isolated from primary wild-type (Wt-p) and immortalized (Wt-i) astrocytes derived from 1- to 2-day-old *Plec*^+*/*+^ and *Plec*^+*/*+^*p53*^*−/−*^ mice, respectively, were subjected to RT-PCR using isoform-specific primers as listed in Additional file 1: Table S2. **A**_**i**_ Expression pattern of plectin isoforms in primary astrocytes. Note that isoforms P1c, P1e, and P1g, which start with the alternative first exons Ex1c, Ex1e, and Ex1g, are the ones most abundantly expressed in astrocytes. **A**_**ii**_ Comparison of isoform P1c, P1e, and P1g expression patterns in primary (Wt-p) versus immortalized (Wt-i) astrocytes. The average for each is plotted with standard deviation error bars drawn. The difference between the means of the two groups was not statistically significant (*P* > 0.05; paired t-test). **B** Fluorescent micrographs of NMO-IgG-labeled plasmalemmal AQP4 aggregates (green) and immunolabeled plectin (red) in a human astrocyte (**B**_**i**_), a U-251 MG cell (**B**_**ii**_), and a *Plec*^+*/*+^*p53*^*−/−*^ astrocyte (**B**_**iii**_). **B**_**iv**_ The percentage of plasmalemmal AQP4 aggregates that colocalize with native plectin is higher in U-251 MG cells as well as *Plec*^+*/*+^*p53*^*−/−*^ astrocytes, compared with human astrocytes (*P < 0.05; one-way ANOVA followed by Dunn’s method). **C** Fluorescent micrographs of U-251 MG (**C**_**i**_), *Plec*^*−/−*^*p53*^*−/−*^ and *Plec*^+/+^*p53*^*−/−*^ astrocytes (**C**_**ii**_, **C**_**iii**,_ respectively), transfected with P1c-mCherry (red) and labeled with NMO-IgG (green). Cells are outlined in white. Arrows point to boxed areas shown enlarged in insets. White masks depict plasmalemmal AQP4 aggregates colocalizing with plectin (pinpointed by arrowheads). Scale bars: 20 µm (enlarged section, 0.5 µm). **C**_**iv**–**vi**_ Statistics showing that P1c’s colocalization with plasmalemmal AQP4 aggregates upon forced expression in U-251 MG cells and in *Plec*^*−/−*^ and *Plec*^+*/*+^ astrocytes is significantly higher compared with that of the other transiently expressed isoforms (P1c/2α3α, P1e, and P1g) (*P < 0.05; one-way ANOVA followed by Dunn’s method). Human astrocytes were isolated from two donors, astrocytes from two *Plec*^*−/−*^*p53*^*−/−*^ and two *Plec*^+*/*+^*p53*^*−/−*^ mice. Experiments were performed in duplicate. The numbers above the boxplots indicate the number of cells analyzed

The N-terminal diversity, generated by alternative splicing of distinct first coding exons into exon 2 of the plectin gene, gives rise to nine plectin isoforms with different N termini that dictate their cellular localization [7, 9, 31]. The expression profiles of the isoforms in some cell types, such as keratinocytes, myocytes, endothelial cells, and Schwann cells, have been established, but this was not the case for astrocytes. Thus, for assessing astrocyte-specific functions of isoforms, we first determined the gene expression levels of isoforms expressed in primary astrocytes isolated from wild-type mice. Our analysis by RT-PCR pointed to P1c as the most abundantly expressed isoform, followed by P1e and P1g (Fig. 5Ai). In a subsequent analysis, we compared the expression levels of these three isoforms in primary versus immortalized (p53-deficient) astrocytes. Primary and immortalized astrocytes showed practically equivalent levels of the three isoforms (Fig. 5Aii), suggesting that p53 deficiency did not affect plectin expression. If confirmed in vivo, these data would be consistent with P1c, P1e, and P1g, playing important roles in CNS functions involving astrocytes.

Next, we tested whether the higher abundance of plasmalemmal AQP4 aggregates of immortalized cells is reflected also in increased colocalization of plectin with these aggregates. To this end, we immunolabeled plectin and plasmalemmal AQP4 in human astrocytes, U-251 MG cells, and immortalized mouse astrocytes *Plec*^+*/*+^*p53*^*−/−*^ (Fig. 5Bi–iii). The analysis revealed that in *Plec*^+*/*+^*p53*^*−/−*^cells and in the U-251 MG cell line, the abundance of plectin at plasmalemmal AQP4 aggregates was ∼2.5 times higher in comparison with human astrocytes (Fig. 5Biv). We then examined which isoform of plectin was primarily targeted to these aggregates. For this, we transfected U-251 MG cells and immortalized astrocytes (*Plec*^*−/−*^*p53*^*−/−*^ and *Plec*^+*/*+^*p53*^*−/−*^) with plasmids encoding fluorescently labeled isoforms P1c, P1e, and P1g, and the P1c variant P1c/2α3α, followed by immunolabeling for plasmalemmal AQP4 aggregates 48 h after transfection (Fig. 5Ci–iii and Additional file 2: Fig. S3). Of all plectin isoforms tested, P1c was by far the most prominently associating with plasmalemmal AQP4 aggregates (Fig. 5Civ–vi).

### Collective cell migration and the dynamics of hypotonicity-induced cell volume changes are impaired in plectin-deficient astrocytes

**Fig. 6:**
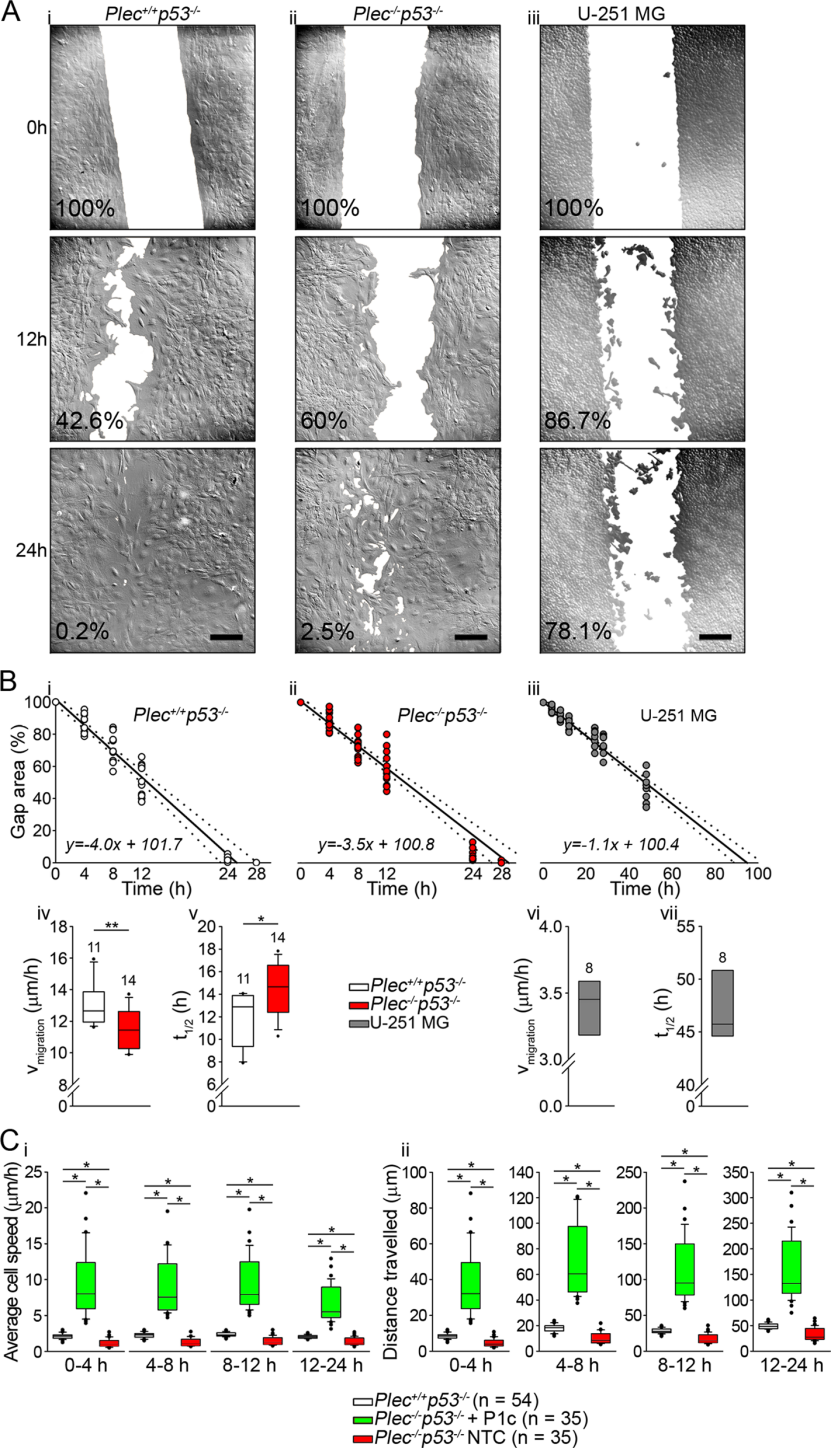
The mobility of astrocytes is plectin dependent. (A) Time-lapse images of *Plec*^+*/*+^*p53*^*−/−*^ (**A**_**i**_) and *Plec*^*−/−*^*p53*^*−/−*^ (**A**_**ii**_) astrocytes, and of U-251 MG GBM cells (**A**_**iii**_) at 0, 12, and 24 h after the initiation of a wound-healing assay. The cell-free (gap) area is depicted in white. The percentages indicate the area of the gap area versus the initial gap in the representative images. Scale bars: 200 µm. **B** The closure of the gap over time is shown for *Plec*^+*/*+^*p53*^*−/−*^ (**B**_**i**_), *Plec*^*−/−*^*p53*^*−/−*^ (**B**_**ii**_), and U-251 MG (**B**_**iii**_) cells. The complete closure of the gap was achieved in 25.2 ± 0.9 h for *Plec*^+*/*+^*p53*^*−/−*^, 29 ± 0.9 h for *Plec*^*−/−*^*p53*^*−/−*^, and 94.7 ± 0.9 h for U-251 MG cells. Solid lines represent the linear regression of the first four time points (0–12 h). Dotted lines indicate confidence limits (α = 0.05). **B**_**iv**,**v**_ The collective migration of *Plec*^+*/*+^*p53*^*−/−*^ astrocytes was faster in comparison with *Plec*^*−/−*^*p53*^*−/−*^ astrocytes (**P < 0.01; Mann–Whitney U test), which was reflected in the shorter time needed for *Plec*^+*/*+^*p53*^*−/−*^ cells to repopulate 50% of the gap (*t*_1/2;_ *P < 0.05; Mann–Whitney U test). **B**_**vi**,**vii**_ The median speed of the collective migration of U-251 MG cells was 3.5 ± 0.1 µm/h, and the median *t*_1/2_ = 45.7 ± 1.1 h. The data were obtained from astrocytes isolated from two *Plec*^+*/*+^*p53*^*−/−*^ and two *Plec*^*−/−*^* p53*^*−/−*^ mice, and from U-251 MG cells. **C** Graphs depict enhanced mobility of *Plec*^*−/−*^*p53*^*−/−*^ astrocytes after forced expression of isoform P1c. Note that the average speed of *Plec*^*−/−*^*p53*^*−/−*^ cells transfected with P1c was higher than that of *Plec*^+*/*+^*p53*^*−/−*^ cells in each time period measured; the speed of non-transfected *Plec*^*−/−*^*p53*^*−/−*^ cells (NTC) was the lowest (**C**_**i**_). In line with the velocity measurements, *Plec*^*−/−*^*p53*^*−/−*^ NTC migrated over the shortest distance (**C**_**ii**_) (*P < 0.05; one-way ANOVA followed by Dunn’s method). The data were obtained from astrocytes isolated from two *Plec*^+*/*+^*p53*^*−/−*^ and two *Plec*^*−/−*^*p53*^*−/−*^ mice, and from U-251 MG cells. Numbers above the boxplots in **B** denote the number of replicates; **C** n denotes the number of cells analyzed

Given the evidence that plasmalemmal AQP4 is involved in GBM migration [15, 16, 47] and that plasmalemmal AQP4 is prominently associated with plectin, we next investigated how plectin affects the migration of astrocytes. For this, we performed a wound-healing assay (Fig. 6A). The results revealed that the speed of the collective migration of *Plec*^*−/−*^*p53*^*−/−*^ astrocytes was reduced by ∼15% compared with that of *Plec*^+*/*+^*p53*^*−/−*^ astrocytes (Fig. 6Bi–iv). The time needed for cells to close 50% of the gap between the two confluent cell populations (*t*_1/2_) was significantly longer for *Plec*^*−/−*^*p53*^*−/−*^ compared with *Plec*^+*/*+^*p53*^*−/−*^ (Fig. 6Bv). To evaluate the contribution of individual plectin isoforms to the mobility of single astrocytes, we performed wound-healing assays with *Plec*^*−/−*^*p53*^*−/−*^astrocytes transfected with plasmids encoding either isoform P1c, P1e, or P1g, and compared their mobility with that of neighboring non-transfected cells and *Plec*^+*/*+^*p53*^*−/−*^ astrocytes. The most prominent increase in speed and travel distance in *Plec*^*−/−*^*p53*^*−/−*^ astrocytes was detected upon forced expression of isoform P1c (Fig. 6C). Nevertheless, *Plec*^*−/−*^*p53*^*−/−*^ astrocytes transfected with plasmids encoding isoform P1e or P1g all showed mobility increases that were at least as high as that of *Plec*^+*/*+^*p53*^*−/−*^ astrocytes (P1e) or higher (P1g) (Additional file 2: Fig. S4). In addition, we measured the collective migration of U-251 MG cells (Fig. 6) and found that it was below that of mouse astrocytes (Fig. 6Bvi, vii). This result was consistent with an earlier study showing decreased migration potential of this type of cells [53].

**Fig. 7:**
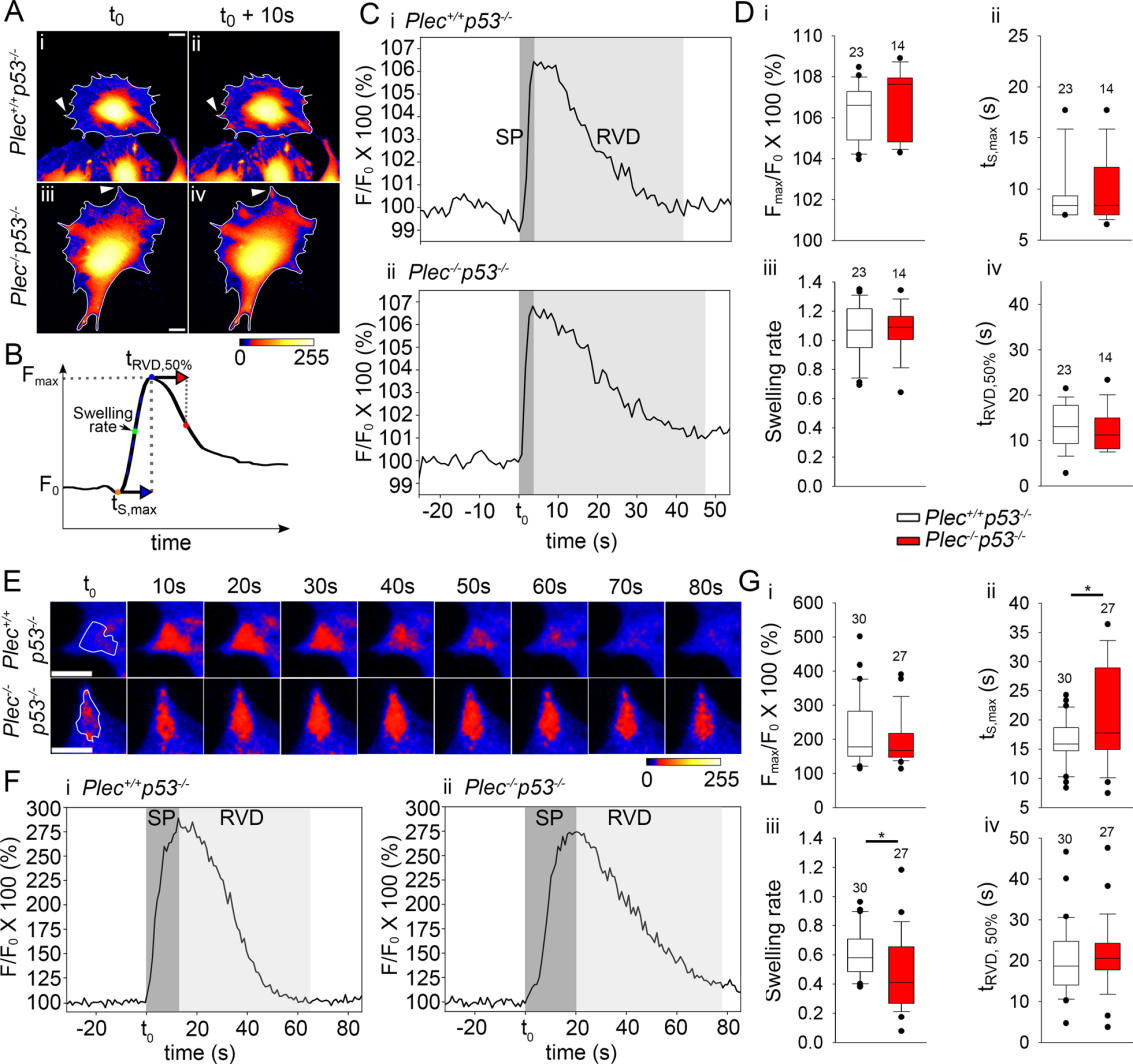
Plectin deficiency leads to local hypo-osmotic shock-induced changes in cell volume. **A** Calcein AM loaded *Plec*^+*/*+^*p53*^*−/−*^ and *Plec*^*−/−*^*p53*^*−/−*^ astrocytes recorded in iso-osmotic (300 mOsm) (**A**_**i**,**iii**_) and in hypo-osmotic (200 mOsm) conditions. The pseudocolored scale of 0–255 corresponds to fluorescence intensity, with cooler colors (blue) representing lower intensity and hotter colors (red) indicating higher intensity (and concentration) of calcein. (A_ii,iv_) Hypo-osmotic conditions triggered an increase in calcein fluorescence. Scale bar: 20 µm. *t*_0_, time point of induction of hypo-osmotic conditions; *t*_0_ + 10 s, time point 10 s after induction of hypo-osmotic conditions. **B** Recording of the fluorescence intensity of calcein over time in single astrocytes, reflecting the parameters of cell volume changes, i.e., *F*_max_ (maximal increase in the volume), *F*_0_ (normalized baseline volume), *t*_s,max_ (swelling duration), the (maximal) swelling rate, and *t*_RVD,50%_ (half-time of RVD). **C** Representative recordings of the relative fluorescence changes (*F*/*F*_0_ × 100%) in *Plec*^+*/*+^*p53*^*−/−*^ (**C**_**i**_) and *Plec*^*−/−*^*p53*^*−/−*^ (**C**_**ii**_) astrocytes. Dark gray denotes the swelling phase (SP); RVD is shaded in light gray. **D** Changes in the whole-cell volume. Note that the maximum relative fluorescence increase (*F*_max_/*F*_0_ × 100%) (**D**_**i**_), *t*_s,max_ (**D**_**ii**_), the swelling rate (**D**_**iii**_), and *t*_RVD,50%_ (**D**_**iv**_) were similar between *Plec*^+*/*+^*p53*^*−/−*^ and *Plec*^*−/−*^*p53*^*−/−*^ astrocytes. **E** Time-lapse sequences of local peripheral regions (LPRs). Two LPRs from (**A**) are shown (marked with arrowheads) displaying changes in the fluorescence intensity of calcein (in pseudocolors) in *Plec*^+*/*+^*p53*^*−/−*^ and *Plec*^*−/−*^*p53*^*−/−*^ astrocytes. Scale bars: 10 µm. **F** Representative recordings of relative cell fluorescence changes (*F*_max_/*F*_0_ × 100%) measured in an LPR of a single *Plec*^+*/*+^*p53*^*−/−*^ (**F**_**i**_) and *Plec*^*−/−*^*p53*^*−/−*^ (**F**_**ii**_) astrocyte (swelling phase (SP) in dark gray, RVD in light gray). **G** Note that the duration of swelling (*t*_S,max_) was increased (**G**_**ii**_) and the swelling rate (**G**_**iii**_) of LPR was reduced in *Plec*^*−/−*^*p53*^*−/−*^ compared with *Plec*^+*/*+^*p53*^*−/−*^ astrocytes (*P < 0.05, Mann–Whitney U test); the maximum relative fluorescence increase (**G**_**i**_) and *t*_RVD,50%_ (**G**_**iv**_) were similar. Numbers above the boxplots denote the number of cells (**D**) and LPRs (**G**) analyzed

To address the role of plectin in the regulation of the cell volume, immortalized *Plec*^+*/*+^*p53*^*−/−*^ and *Plec*^*−/−*^*p53*^*−/−*^ astrocytes were loaded with Calcein AM and exposed to a hypo-osmolar solution (200 mOsm) (Fig. 7A). When the whole-cell volume of astrocytes was measured, there were no significant changes in any of the measured parameters, i.e. amplitude (*F*_max_*/F*_0_ × 100%), swelling rate, maximal swelling duration (*t*_S,max_), and half-time of the RVD (*t*_RVD,50%_) between both phenotypes observed (Fig. 7B–D). However, the local peripheral regions (LPRs) of astrocytes displayed fluorescence changes in calcein, coinciding with the formation of bleb-like protrusions that were simultaneously observed in a DIC image (Additional file 3: Video S1). Differences in volume dynamics of LPRs were noticeable between *Plec*^+*/*+^*p53*^*−/−*^ and *Plec*^*−/−*^*p53*^*−/−*^astrocytes (Fig. 7E–G). In particular, *t*_S,max_ of LPR was prolonged by ∼10% (Fig. 7E, F), and the swelling rate of LPRs was reduced by ∼25% in *Plec*^*−/−*^*p53*^*−/−*^ in comparison with *Plec*^+*/*+^*p53*^*−/−*^ astrocytes. The maximal volume increase (*F*_max_*/F*_0_ × 100) and *t*_RVD,50%_ were similar in both phenotypes (Fig. 7G).

These data show that the absence of plectin impairs the migration of astrocytes and significantly affects the dynamics of cell volume changes at the cell periphery.

### U-251 MG and T98G cells abundantly express plectin at their surface

**Fig. 8:**
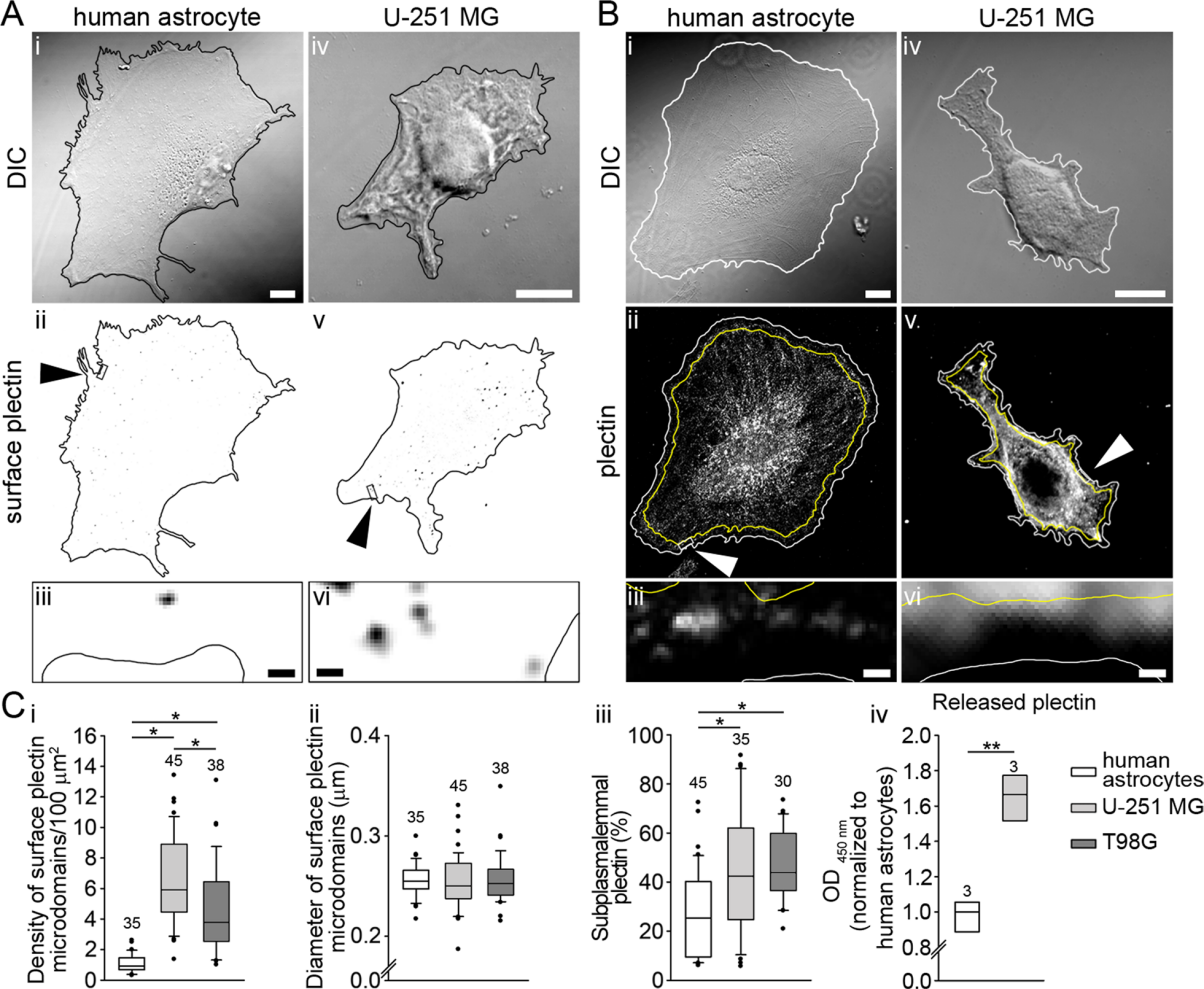
U-251 MG and T98G cells express more surface plectin microdomains and have more subplasmalemmal plectin than human astrocytes. **A**_**i**–**vi**_ DIC images of a human astrocyte (**A**_**i**–**iii**_) and of a U-251 MG cell (**A**_**iv**–**vi**_) with the corresponding inverted fluorescent micrographs and enlarged sections below depicting surface plectin microdomains. Cell outlines are shown in black; surface plectin microdomains in inverted fluorescent micrographs are displayed as black puncta. Arrowheads in **A**_**ii**,**v**_ point to demarcated areas that are enlarged in panels below (**A**_**iii**,**vi**_). Scale bars: 10 µm (enlarged section, 0.5 µm). **B**_**i**–**vi**_ Vertically aligned DIC images of a human astrocyte (**B**_**i**–**iii**_) and a U-251 MG cell (**B**_**iv**–**vi**_) are shown with the corresponding inverted fluorescent micrographs of immunolabeled plectin and enlarged sections below depicting intracellular plectin. Rectangular regions in (**B**_**ii**,**v**_) demarcated in white and indicated by arrowheads are enlarged below in **B**_**iii**,**vi**_. Cell boundaries are outlined in white and subplasmalemmal areas (the area between the cell outline and the line calculated by reducing the cell area by 10%) are outlined in yellow. Scale bars: 10 µm (enlarged sections, 0.5 µm). **C**_**i**_ The density of surface plectin microdomains is lower in human astrocytes compared with U-251 MG and T98G cells (*P < 0.05; one-way ANOVA followed by Dunn’s method), whereas diameters of surface plectin microdomains (**C**_**ii**_) are similar among all cell types (*P* = 0.623; Kruskal–Wallis test). **C**_**iii**_ Subplasmalemmal distribution of plectin in the cytoplasm of human astrocytes, U251 MG and T98G cells. Note that subplasmalemmal plectin is ~ 2 times more abundant in U-251 MG and T98G cells than in human astrocytes (*P < 0.05 one-way ANOVA followed by Dunn’s method). **C**_**iv**_ Released plectin from cells is depicted as optical density (OD) at 450 nm using rabbit anti-plectin antiserum #9. U-251 MG cells release approximately 1.5 times more plectin as human astrocytes (**P < 0.01; Student’s t test). Data were obtained from human astrocytes isolated from two donors. Experiments were performed in triplicate. In panels **C**_**i**–**iii**_ the numbers above the boxplots are the number of cells analyzed, while in the panel **C**_**iv**_ the numbers represent the number of independent samples

In several types of cancer plectin is upregulated and its intracellular localization is altered. Remarkably, the protein has been identified as a specific and abundant cell surface target in pancreatic ductal carcinoma and several other cancers, including ovarian, lung, and prostate cancer. Thus plectin has emerged as a biomarker for a number of cancers and it became a candidate for targeting therapies in multiple malignancies [33, 34, 36–40]. Therefore, we examined the abundance of cell surface plectin among U-251 MG and T98G GBM cell lines and human astrocytes. For the labeling of live cells with pan-plectin antibody, we followed the same protocol as for the labeling of plasmalemmal AQP4 aggregates. Puncta, indicating the presence of surface plectin microdomains in human astrocytes and U-251 MG cells are depicted in Fig. 8A and the subplasmalemmal localization of plectin in these cells is delineated in Fig. 8B. Substantially more surface plectin microdomains were present in U-251 MG and T98G cells, six and four times more, respectively, compared with human astrocytes (Fig. 8Ci), while the diameter of surface plectin microdomains did not differ among these cell types (Fig. 8Cii). Moreover, the subplasmalemmal localization of plectin in U-251 MG and T98G cells was significantly more prominent than in human astrocytes (Fig. 8Ciii) and the released plectin in the extracellular medium was 1.5 times more abundant in U-251 MG cells as in human astrocytes (Fig. 8C). In summary, in the GBM cell lines, plectin conspicuously relocates toward the cell periphery and the cell surface. Furthermore, the release of plectin from the GBM cell line into the extracellular space is enhanced in comparison with primary astrocytes. Hence, plectin’s accessibility at the cell surface could be explored as a diagnostic marker and potential therapeutic target.

## Discussion

In the present study, we demonstrate that the expression and localization of plectin in brain tumors correlate with those of AQP4 and that the abundance of plectin on the extracellular leaflet of the plasmalemma is increased in glioblastoma cells. Both of these observations have the potential to become useful in identifying GBMs and in defining a direct therapeutic target. We identified the isoforms of plectin that are most abundantly expressed in astrocytes and describe their localization with regard to plasmalemmal AQP4 aggregates. Our results reveal that plectin regulates the size and the abundance of plasmalemmal AQP4 aggregates and that it affects the migratory potential of GBM cells through mechanisms involving cytoskeletal rearrangements and modulation of cytoplasmic volume dynamics at the astrocyte periphery.

The upregulation of AQP4 expression in brain tumors has been proposed to modulate human glioma cell migration, cell invasion, and cerebral edema [47, 54–56]. Previous studies have shown that high-grade tumors have higher expression of AQP4 than healthy brain tissue and low-grade tumors [55]. Our study is the first to reveal a positive correlation between PLEC and AQP4 expression in GBM. We speculate that the observed correlation between increased expression of AQP4 and plectin might be associated with the reactive phenotype of astrocytes. Of note, it has been reported that, similar to IFs, plectin is upregulated in reactive astrocytes [57, 58].

Moreover, we found that plectin and AQP4 colocalize in GBM samples, suggesting that the two proteins might be functionally linked. We could also show that plectin is colocalized with plasmalemmal AQP4 aggregates to a higher degree than GFAP, an isoform of which (GFAP-δ) has previously been proposed as a candidate GBM biomarker [59]. The isoform ratio GFAP-δ/GFAP-α correlates with the expression of MAPK phosphatase 2, which affects cell migration, invasion, and proliferation, and exhibits high expression in GBMs with poor prognosis [59, 60]. It is of interest in this context that we found the codistribution of plectin with AQP4 to be higher in GBMs with a low PI and high invasiveness compared with GBMs with high PI and low invasiveness.

Plectin’s ability to promote cell proliferation and invasion was previously demonstrated in pancreatic ductal adenocarcinoma, non-small-cell lung cancer, high-grade epithelial ovarian cancer, prostate cancer, and head and neck squamous cell carcinoma [20]. However, to the best of our knowledge, the effect of plectin on GBM migration has never been studied. Here, we addressed this issue by (1) identifying which plectin isoforms are expressed in astrocytes, (2) investigating if plectin affects the distribution of plasmalemmal AQP4 aggregates that have been linked to altered migration speed of GBM cells, and (3) measuring the velocity of collective migration of astrocytes expressing, or not expressing, plectin. We demonstrated that in astrocytes, P1c, P1e, and P1g are the most abundantly expressed plectin isoforms, in agreement with the general expression pattern of plectin isoforms in the CNS [9]. Of the three major isoforms identified in astrocytes, P1c was the one predominantly found associated with plasmalemmal AQP4 aggregates. Whether this indicates an isoform-specific interaction mechanism or just reflects that P1c is the isoform most abundantly expressed in astrocytes remains to be clarified.

Previous studies showed that fewer and smaller OAPs were observed in glioma cells with higher migratory and invasive capacity, and an increased number of OAPs was proposed to promote cell adhesion of GBM cells [16, 61]. Our results show that U-251 MG cells, which have been confirmed to express AQP4 [62], express more plasmalemmal AQP4 aggregates compared with human astrocytes, even if smaller in size. These results are in line with the expected higher migratory speed of GBM cells, which express smaller OAPs [47]. The size of plasmalemmal AQP4 aggregates, which according to the literature ranges from 150 to 550 nm [63], depends on many factors, among which the ratio between the two major AQP4 isoforms, M1 and M23, has been proposed to play a decisive role, such that a higher M1/M23 ratio leads to smaller OAPs [16, 54, 61]. Thus, we assume that the smaller size of plasmalemmal AQP4 aggregates observed in U-251 MG cells is also due to an increased M1/M23 ratio, even though this point was not specifically addressed in our study. Of note, the patient’s plasma NMO-IgGs represent a group of antibodies that likely recognize different epitopes of the extracellular region of AQP4. NMO-IgGs preferentially bind to AQP4 incorporated into OAPs, although they can also recognize the individual AQP4 tetramers [13, 63]. Considering the size of individual OAPs and the limitations in the resolution of confocal microscopy, our fluorescent plasmalemmal AQP4 aggregates may represent individual OAPs, clusters of OAPs, and/or individual AQP4 tetramers.

Positioning of plasmalemmal AQP4 aggregates is also mediated by interactions between AQP4 tetramers and several extracellular and intracellular proteins, including agrin, laminin, and the dystrophin–dystroglycan complex which binds to F-actin [16, 48, 49]. Plectin is involved in the regulation of AF dynamics [64], and, as shown for skeletal muscle, it interacts directly with ß-dystroglycan via multiple domains and with dystrophin/utrophin via its ABD [52]; binding to the ß-dystroglycan/utrophin complex has also been shown for Schwann cells [17]. Thus, we hypothesized that the dynamics of plasmalemmal AQP4 aggregates may be modulated by plectin, perhaps indirectly, via the ß-dystroglycan/dystrophin complex. Our findings demonstrate that, in the presence of functional plectin, astrocytes expressed higher levels of plasmalemmal AQP4 aggregates that were larger in comparison with astrocytes devoid of plectin. Furthermore, the distribution and bundling of AFs was affected by the expression of plectin, such that in plectin-deficient astrocytes, AFs retracted from the subplasmalemmal region. Our results are in line with a previous report demonstrating the interrelationship between AQP4 expression and organization of AFs in astrocytes, where knocking down AQP4 expression resulted in depolymerization and rearrangement of AFs [64]. While supporting the concept of AQP4-AF interdependence, our results identify plectin as a novel partner that through its interaction potential with AFs and IFs can be crucial for the positioning of plasmalemmal AQP4 aggregates. A proposed working model (adapted from ref [48]) reflecting this situation is shown as (Additional file 2: Fig. S5). The validation of this model requires further investigation, where studies involving AQP4 knockout astrocytes could offer crucial information.

Plectin is an important cytolinker of the cytoskeleton, mediating the crosstalk of cytoskeletal filament systems among each other as well as with macromolecular assemblies, critical for cell migration, such as focal adhesions [31]. Therefore, it is not surprising that the depletion of plectin diminished the migratory speed of astrocytes. A similar phenotype was reported for fibroblasts [65]. Our results are also in line with previous studies demonstrating that plectin, together with IFs, is crucial for the organization and dynamics of the actomyosin network [31, 66, 67]. In their study on astrocytes, De Pascalis et al. [66] reduced plectin expression by siRNA, which impaired the collective migration of cells. Our results with primary and plectin-null astrocytes confirm and extend their findings by offering first insights into astrocyte-specific functions of plectin isoforms, in particular of P1c, the most abundantly expressed astrocytic variant.

In several cell types, including myoblasts, keratinocytes, and alveolar epithelial cells, plectin was found to bind to β-DG [17, 18, 68]. In Schwann cells, where plectin interacts directly with β-DG and vimentin, ablation of plectin abrogated the tight association of the dystroglycan complex with VFs, turning VFs into a sparser network and leading to myelin sheath deformations [17]. We show now that in plectin-deficient astrocytes, the VF network is also more bundled and sparser and extends more into the region below the plasma membrane. Bundling of VFs has been demonstrated to inhibit migration and proliferation of simvastatin-treated cancer cells, indicating that a flexible VF network promotes cell migration and that bundling of VFs perturbs the invasive properties of cells [69].

OAPs have been linked to alterations in migration speed [47]. AQP4 has adhesive properties and is assumed to facilitate astrocyte mobility by permitting rapid volume changes at the leading edges of migrating astrocytes [70]. In particular, disintegration of plasmalemmal AQP4 aggregates and an increased amount of individually expressed freely mobile M1-AQP4 isoform in the plasma membrane diffusing into rapidly extending lamellipodial regions could support cell migration [61]. However, there might be undiscovered mechanisms, beyond the ratio between M1 and M23 isoforms of AQP4, that regulate the assembly of the supramolecular plasmalemmal AQP4 aggregates [70]. A possible mechanism underlying the modulation of plasmalemmal AQP4 aggregates may be mediated by plectin’s crosstalk with AF and VF networks and/or via plectin’s direct interaction with the DG complex.

In a study on a subtype of glial radial cells, AQP4 and VFs were reported to colocalize in 100% of the cellular processes [71]. The interaction between AQP4 and VFs is mediated by a molecular complex, part of which is β-DG [49]. Upon brain-selective deletion of the β-DG gene, most of the superficial AQP4 molecules do not form OAPs, and AQP4 expression in perivascular endfeet is compromised [72]. Plectin contributes to stabilization of the DG complex by anchoring β-DG directly to the VF network [17]. Given that the lack of plectin expression in astrocytes induces bundling and redistribution of VFs and leads to a reduction in the size and abundance of plasmalemmal AQP4 aggregates, we propose that the migratory potential of GBM cells (in which we found the expression of plectin to correlate with that of AQP4) is plectin dependent.

Besides migration of GBM cells within the CNS of patients with GBM, the development of edema can also pose a serious complication [73]. Based on the observation that AQP4 knockout astrocytes exhibit slower kinetics of cell swelling, AQP4 has been proposed to be a major regulator of CNS water homeostasis [74]. Moreover, edema has also been linked to increased levels of AQP4 [49, 55], in part because in brain tumors, water moves into the brain through a leaky blood–brain barrier via a bulk fluid flow mechanism [13]. Combining previous findings suggesting that (1) cell-swelling kinetics of astrocytes in hypo-osmotic conditions is affected by the expression of M1 (AQP4a), M23 (AQP4c), and AQP4e isoforms [38, 74, 75] and (2) several actin-binding proteins contribute to the process of cell swelling [76], we postulate that plectin contributes, at least indirectly, to the swelling kinetics of immortalized astrocytes. By employing calcein measurements, we observed no significant changes in the dynamics of the whole-cell volume between plectin-expressing and plectin-deficient immortalized astrocytes exposed to a hypo-osmolar solution. However, our measurements revealed a prolonged duration of swelling and a reduced swelling rate in hypo-osmotic conditions in localized regions near the cell periphery of plectin-deficient immortalized astrocytes. Local thickening of the cell resulted in bleb-like protrusions. The mechanisms involved in the regulation of bleb expansion and retraction involve cytoskeletal elements (plectin likely plays an important role in this process), calcium, and cytoplasmic fluidity [77]. AQP4, as the primary water channel in astrocytes, is predominantly located in the plasmalemma within OAPs which facilitate relatively rapid local water transport [48]. Alterations in the abundance of plasmalemmal AQP4 aggregates within blebs, as observed in astrocytes of both, *Plec*^+*/*+^ and *Plec*^*−/−*^ genotypes, potentially might result in localized changes in cell volume. Whether a mechanism such as this indeed exists remains to be addressed in future studies.

In line with previous studies on astrocytes, the RVD immediately followed the swelling phase measured in the whole cell and in the LPR [38, 75]. Of note, peripheral position of LPRs in cultured astrocytes was suggested to roughly correspond to perivascular AQP4-enriched endfeet in vivo [75]. Thus, the observed difference in LPR swelling of plectin-expressing and plectin-deficient astrocytes points towards an important role of plectin in brain edema formation. This would be consistent with its previously suggested role in the maintenance of the structural and functional integrity of the blood–brain barrier and pial surface [11, 78].

In light of plectin’s emerging role as a potential biomarker and therapeutic target, based on its cell surface localization in several cancers [20, 21, 23–27], we investigated whether plectin should also be considered as a biomarker of GBM. Showing that U-251 MG and T98G cells express higher levels of surface plectin in comparison with human astrocytes, our data do imply that plectin could be used as a biomarker and drug delivery agent in GBM. The detection of plectin at the surface of U-251 MG and T98G cells is in line with the strongly increased plectin-specific staining of the plasma membrane in pancreatic and other types of cancer, including bile duct cholangiocarcinoma, lung adenocarcinoma, lung squamous cell carcinoma, ovarian cancer, and intestinal type stomach cancer [79]. Also, in certain types of cancers, the bulk cytoplasmic distribution of plectin is altered, predominantly redistributing toward the plasma membrane. This observation was noticed, for example, in serous cystadenocarcinomas and clear cell epithelial ovarian cancer [21]. In line with these findings, we detected a higher proportion of plectin near the plasma membrane of U-251 MG and T98G cells compared with human astrocytes. Furthermore, in agreement with the reported release of plectin from various pancreatic ductal adenocarcinoma cell lines [22], we observed an increased release of plectin from U-251 MG cells. While the release of plectin from pancreatic ductal adenocarcinoma cell lines has been confirmed to occur via exosomes [22], the mechanism of plectin release from astrocytes and GBMs remains to be elucidated. For validating plectin’s potential as a biomarker of GBMs, it will be essential to investigate its re-localization within the cytoplasm, assess its surface expression, and measure the direct release of plectin in biopsy specimens obtained from GBMs and lower-grade astrocytomas.

## Conclusions

Our study shows that plectin colocalizes with AQP4 and plasmalemmal AQP4 aggregates and regulates their abundance and size. We found that astrocytes predominantly express the plectin isoforms P1c, P1e, and P1g, with P1c being prominently associated with plasmalemmal AQP4 aggregates and strongly affecting astrocyte mobility. Cells lacking plectin show greatly reduced mobility. Moreover, we found that the absence of plectin and the consequent remodeling of the subplasmalemmal cytoskeleton impair the peripheral swelling dynamics of astrocytes. This suggests a potentially vital role of plectin in ameliorating tumor-induced edema. This report is the first to demonstrate the redistribution of plectin toward the plasmalemma and the cell surface, along with the release of plectin from GBM cells. These findings highlight the potential of plectin as both a biomarker of GBM and a contributor to GBM migration. Hence, our study opens new possibilities for the more specific identification of GBMs by targeting plectin, which may represent a novel therapeutic strategy to prevent or ameliorate GBM invasiveness.

### Supplementary Information

**Additional file 1: Table S1.** Percentage of cells expressing astrocyte markers. **Table S2.** Primers used for RT-PCR.

**Additional file 2: Fig. S1.** NMO-IgG (anti-AQP-4 antibodies) confirmed in a NMO patient serum. **Fig. S2.** PLEC/AQP4 and PLEC/GFAP colocalization assessed in a biopsy sample obtained from a GBM patient. **Fig. S3.** Colocalization of plasmalemmal AQP4 aggregates (pAQP4) with plectin isoforms P1c/2α3α, P1e and P1g in U-251 MG cells, and in* Plec*^−/−^*p53*^−/−^ and *Plec* ^+ / + ^*p53*^−/−^ astrocytes. **Fig. S4.** Forced expression of isoforms P1e and P1g leads to enhanced mobility of plectin-null astrocytes. **Fig. S5.** Working model depicting interactions between plectin, the dystrophin-glycoprotein complex and AQP4 in astrocytes.

**Additional file 3: Video S1.** In astrocytes loaded with Calcein AM, an increase in the fluorescence intensity of calcein is observed in local peripheral regions of cells after exposure to a hypo-osmolar solution (200 mOsm). These changes in fluorescence intensity indicate locally observed alterations in the volume dynamics of peripheral cell regions.

## Data Availability

The datasets used and/or analyzed during the current study are available from the corresponding author on reasonable request.

## Abbreviations

AF: Actin filament
AQP4: Aquaporin 4
BCA: Bicinchoninic acid assay
BSA: Bovine serum albumin
CNS: Central nervous system
DIC: Differential interference contrast
DG: Dystroglycan
ELISA: Enzyme-linked immunosorbent assay
FBS: Fetal bovine serum
GBM: Glioblastoma
GFAP: Glial fibrillary acidic protein
HC: Healthy control
IDH1: Isocitrate dehydrogenase (NADP(+))
IF: Intermediate filament
Ki-67: Non-histone nuclear protein
LPR: Local peripheral region
MT: Microtubule
NMO-IgG: Neuromyelitis optica immunoglobulin G antibodies (anti-AQP4 antibodies)
NSC: Neural stem cell
OAPs: Orthogonal array of particles
PBS: Phosphate-buffered saline
PDL: Poly-d-lysine
PI: Proliferative index
PLEC: Plectin
PN: Perinuclear
RT-PCR: Real-time polymerase chain reaction
RVD: Regulatory volume decrease
RT: Room temperature
SP: Swelling phase
SPL: Subplasmalemmal
TMB: TMB (3,3ʹ,5,5ʹ-Tetramethylbenzidine) is a substrate for horseradish peroxidase
VF: Vimentin filament
