# Supplementary material for: Plectin plays a role in the migration and volume regulation of astrocytes: a potential biomarker of glioblastoma
Source: J Biomed Sci. 2024 Jan 23;31:14. doi: 10.1186/s12929-024-01002-z (PMC10807171; doi:10.1186/s12929-024-01002-z)
Supplement: Supplementary file 1 — Additional file 1: Table S1. Percentage of cells expressing astrocyte markers. Table S2. Primers used for RT-PCR. [file 12929_2024_1002_MOESM1_ESM.pdf]

Additional information

Table S1 Percentage of cells expressing astrocyte markers

Cell type[1)]	% of labeled cells[2)]				
	**GFAP[3)]**	**AQP4**	**ALDH1L1**	**GS**	**S100β**
Plec$^{+/+}$	99.3 ± 0.7[4)]	98.1 ± 1.3	99.1 ± 0.9	98.5 ± 0.4	91.1 ± 1.0
Plec$^{-/-}$	98.9 ± 1.1	98.8 ± 0.8	99.1 ± 0.9	90.5 ± 0.7	90.1 ± 0.9
Plec$^{+/+}$*p53*$^{-/-}$	98.6 ± 0.6	99.0 ± 0.8	99.2 ± 0.5	98.2 ± 0.5	90.3 ± 1.1
Plec$^{-/-}$*p53*$^{-/-}$	99.5 ± 0.5	99.6 ± 0.4	98.5 ± 1.0	91.3 ± 0.9	90.9 ± 2.3
Human astrocyte	99.2 ± 0.8	100.0 ± 0.0	99.0 ± 0.7		
U-251 MG	99.2 ± 0.8	98.2 ± 0.8	100.0 ± 0.0		

[1)] *Plec*$^{+/+}$, primary mouse astrocytes expressing plectin; *Plec*$^{-/-}$, primary mouse astrocytes lacking plectin expression; *Plec*$^{+/+}$*p53*$^{-/-}$, immortalized mouse astrocytes expressing plectin; *Plec*$^{-/-}$*p53*$^{-/-}$, immortalized mouse astrocytes lacking plectin expression; U-251 MG, permanent cell line derived from a human malignant GBM.

[2)] % reflects the percentage of cells expressing a particular astrocyte marker based on all cells in a field of view.

[3)] Astrocyte markers: GFAP, glial fibrillary acidic protein; AQP4, aquaporin 4; ALDH1L1 aldehyde dehydrogenase 1 family member L1; GS, glutamine synthetase; S100β, S100 calcium-binding protein β.

[4)] Data obtained from triplicates per cell type are represented as mean ± standard error

Table S2 Primers used for RT-PCR

Amplicon[1)]	Forward Primer (5'→3')	Reverse Primer (5'→3')
Exon 1 ↔ Exon 2	CTTGTGCTTAGAACCGGAGC	ACCTGTAGATTGGTGACGCC
Exon 1a ↔ Exon 2	GGTAGCAAGAGAACCAGCTCA	AGGTGTTTGTTGACCCACTTG
Exon 1b ↔ Exon 2	TGGTAGTCGTGGGTCATGTTGTC	AGGTGTTTGTTGACCCACTTG
Exon 1c ↔ Exon 2	AAGTGGAGGTGGTTCTGTGG	AGGTGTTTGTTGACCCACTTG
Exon 1d ↔ Exon 2	GAAGATCGTGCCCGATGAA	AGGTGTTTGTTGACCCACTTG
Exon 1e ↔ Exon 2	ACGAGATCAGCTCCCTCAAA	AGGTGTTTGTTGACCCACTTG
Exon 1f ↔ Exon 2	CCGACGAACAGGATTTCATC	AGGTGTTTGTTGACCCACTTG
Exon 1g ↔ Exon 2	GGCTACCTCTACGGGCAACT	AGGTGTTTGTTGACCCACTTG
HPRT1[2)]	CAGGCCAGACTTTGTTGGAT	TTGCGCTCATCTTAGGCTTT
GAPDH	CACTCACGGCAAATTCAACG	ACACCAGTAGACTCCACGAC

[1)] Amplicons corresponding to plectin's alternative first exons (1-1g) to exon 2.

[2)] Internal controls: HPRT1, hypoxanthine guanine phosphoribosyl transferase 1; GAPDH, glyceraldehyde-3-phosphate dehydrogenase.
