# Supplementary material for: Plectin plays a role in the migration and volume regulation of astrocytes: a potential biomarker of glioblastoma
Source: J Biomed Sci. 2024 Jan 23;31:14. doi: 10.1186/s12929-024-01002-z (PMC10807171; doi:10.1186/s12929-024-01002-z)
Supplement: Supplementary file 2 — Additional file 2: Fig. S1. NMO-IgG (anti-AQP-4 antibodies) confirmed in a NMO patient serum. Fig. S2. PLEC/AQP4 and PLEC/GFAP colocalization assessed in a biopsy sample obtained from a GBM patient. Fig. S3. Colocalization of plasmalemmal AQP4 aggregates (pAQP4) with plectin isoforms P1c/2α3α, P1e and P1g in U-251 MG cells, and in Plec−/−p53−/− and Plec + / + p53−/− astrocytes. Fig. S4. Forced expression of isoforms P1e and P1g leads to enhanced mobility of plectin-null astrocytes. Fig. S5. Working model depicting interactions between plectin, the dystrophin-glycoprotein complex and AQP4 in astrocytes. [file 12929_2024_1002_MOESM2_ESM.pdf]

Additional information

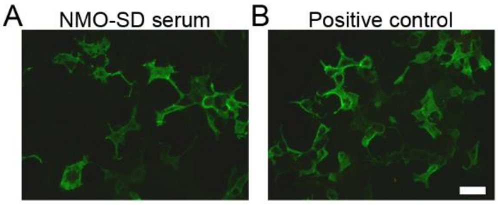

Fig. S1 NMO-IgG (anti-AQP-4 antibodies) confirmed in a NMO patient serum. An indirect immunofluorescence test for NMO-IgG (EUROIMMUN Medizinische Labordiagnostika AG, Lübeck, Germany) was used to detect the presence of antibodies in the serum of a patient with neuromyelitis optica spectrum disorder (NMOSD). (A, B) AQP4-transfected human embryonic kidney 293 (HEK293) cell line incubated with a 1:100 dilution of the patient's serum (A), or a 1:100 dilution of anti-AQP4 positive control sample (B). Images were recorded with the LED fluorescence microscope EUROStar III Plus equipped with a 488 nm excitation filter, a 510 nm color separator and a 520 nm blocking filter (EUROIMMUN Medizinische Labordiagnostika AG).

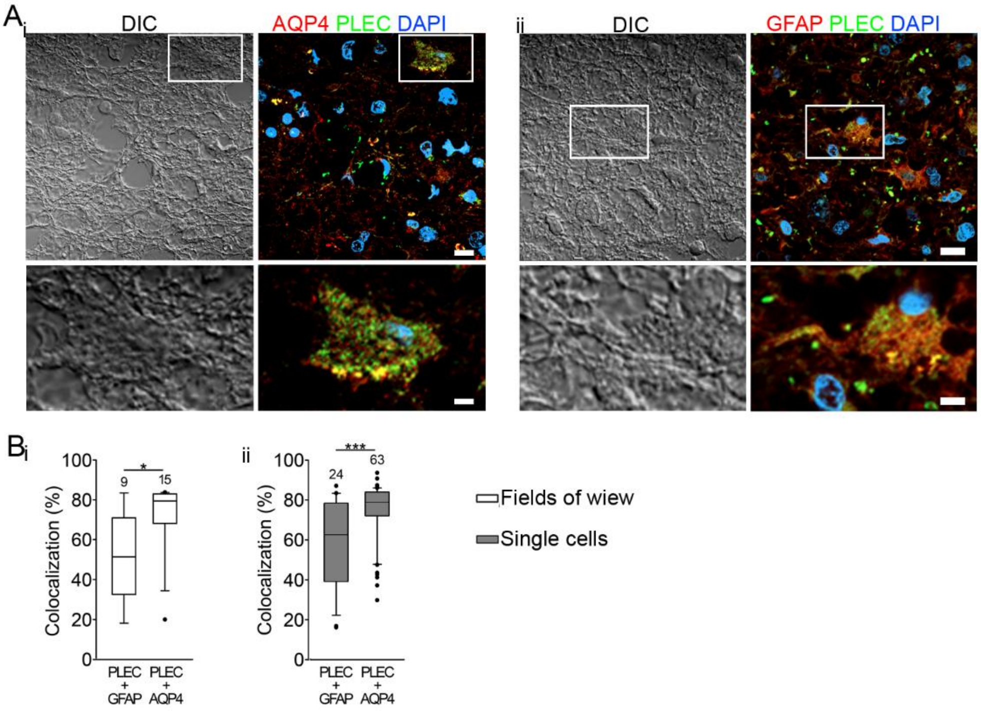

Fig. S2 PLEC/AQP4 and PLEC/GFAP colocalization assessed in a biopsy sample obtained from a GBM patient. (A_i) DIC image and the corresponding fluorescence micrograph depicting NMO-IgG-immunolabeled AQP4, immunolabeled PLEC, and DAPI-labeled nuclei. (A_{ii}) DIC image and the corresponding overlay of fluorescence micrograph depicting immunolabeled GFAP, immunolabeled PLEC and DAPI-labeled nuclei. Yellow color in overlays indicates colocalization of AQP4 with PLEC (A_i) and of GFAP with PLEC (A_{ii}), respectively. Astrocytes within rectangular areas are enlarged below. Scale bars: 10 µm (inset, 3 µm). (B) The colocalization of PLEC with AQP4 is higher than that with GFAP, when the analysis was performed of the entire fields of view (B_i) and of individual cells (B_{ii}) (*$P < 0.05$, ***$P < 0.001$; Mann-Whitney U test). Numbers above the boxplots correspond to the number of fields of view and the number of cells analyzed, respectively.

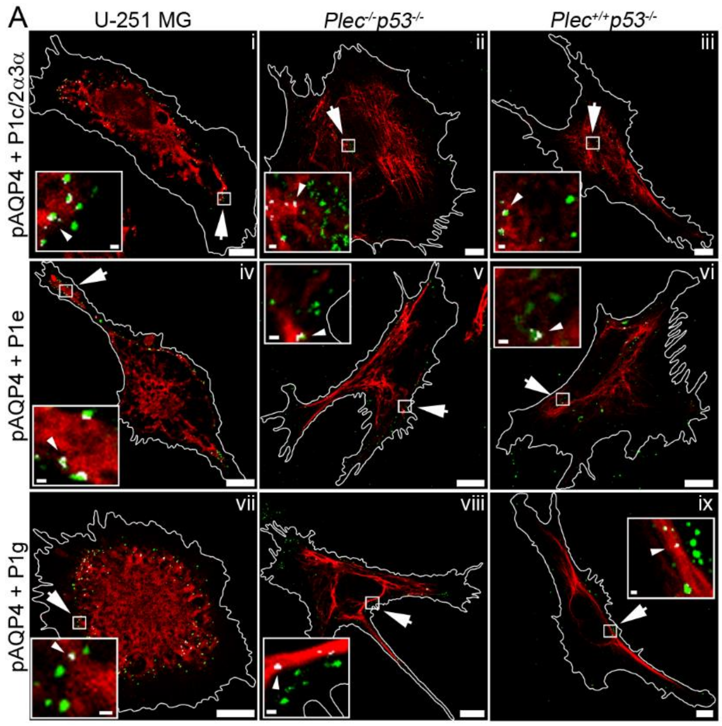

Fig. S3 Colocalization of plasmalemmal AQP4 aggregates (pAQP4) with plectin isoforms P1c/2α3α, P1e and P1g in U-251 MG cells, and in *Plec*$^{-/-}$*p53*$^{-/-}$ and *Plec*$^{+/+}$*p53*$^{-/-}$ astrocytes. Shown are fluorescent micrographs of NMO-IgG-labeled pAQP4 in cells transfected with P1c/2α3α ($A_{i\text{-}iii}$), P1e ($A_{iv\text{-}vi}$), and P1g ($A_{vii\text{-}ix}$). Cells are outlined in white. Arrows point to demarcated boxed areas enlarged in insets. The colocalization masks (white puncta) in insets are pinpointed by arrowheads. Scale bars: 10 µm (enlarged areas, 0.5 µm). Astrocytes isolated from two *Plec*$^{-/-}$*p53*$^{-/-}$ and two *Plec*$^{+/+}$*p53*$^{-/-}$ mice were used for data collection. Experiments were performed in duplicates.

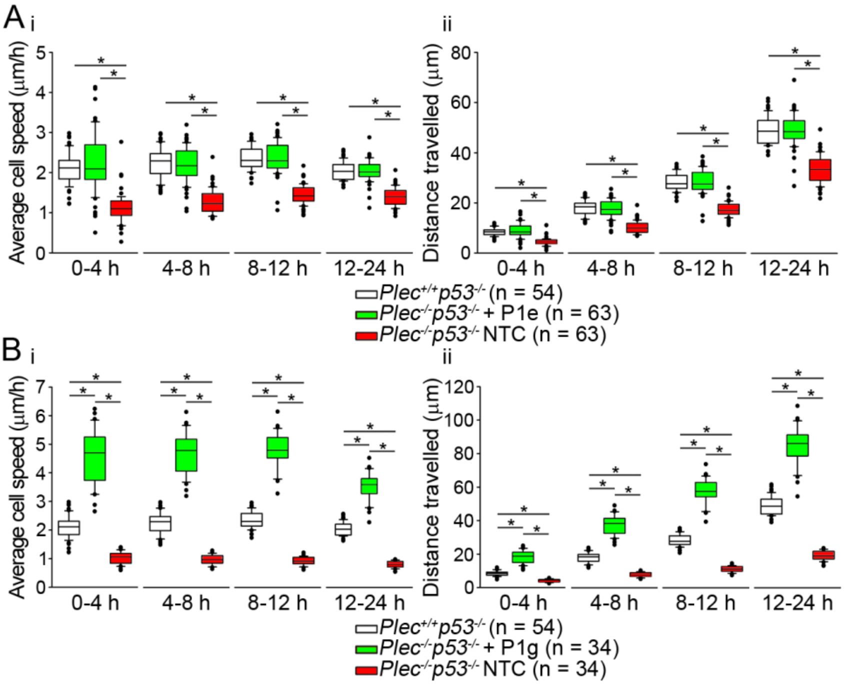

Fig. S4 Forced expression of isoforms P1e and P1g leads to enhanced mobility of plectin-null astrocytes. (A_i) The average speed of *Plec*$^{-/-}$*p53*$^{-/-}$ cells transfected with P1e was similar to the speed of *Plec*$^{+/+}$*p53*$^{-/-}$ cells in each time period measured; the average speed of non-transfected cells (NTC) was always the lowest. The average travel distance was in line with the measurements of the speed, with *Plec*$^{-/-}$*p53*$^{-/-}$ NTCs traveling the shortest distance (A_{ii}) (**P* < 0.05; One-way ANOVA followed by Dunn's Method). (B_i) In each time period measured, the average speed of *Plec*$^{-/-}$*p53*$^{-/-}$ cells transfected with P1g was higher than the speed of *Plec*$^{+/+}$*p53*$^{-/-}$ cells, while the average speed of NTCs was the lowest. The average distances traveled were in line with the velocity measurements, with *Plec*$^{-/-}$*p53*$^{-/-}$ NTC migrating over the shortest distances (B_{ii}) (**P* < 0.05; One-way ANOVA followed by Dunn's Method). The data were obtained from astrocytes isolated from two *Plec*$^{+/+}$*p53*$^{-/-}$ and two *Plec*$^{-/-}$ *p53*$^{-/-}$ mice. Numbers in brackets denote the number of cells analyzed.

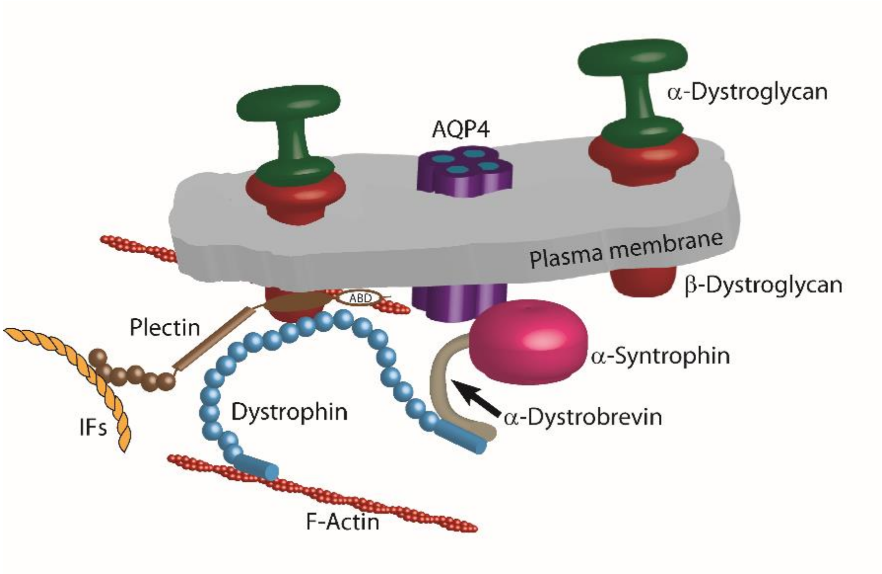

Fig. S5 Working model depicting interactions between plectin, the dystrophin-glycoprotein complex and AQP4 in astrocytes. The schematic includes the plectin molecule, which interacts with β-dystroglycan and dystrophin as part of the dystrophin–glycoprotein complex, actin filaments (F-actin) and intermediate filaments (IFs). The dystrophin–glycoprotein complex is represented by the integral membrane proteins α- and β-dystroglycan, the actin-binding protein dystrophin, α-dystrobrevin, and α-syntrophin. The AQP4 channel is connected to the dystrophin-glycoprotein complex likely via α-syntrophin which, in turn, is linked to dystrophin via α-dystrobrevin. In conclusion, plectin may represent an important stabilizing partner of dystrophin-glycoprotein complex and AQP4; though, direct interactions between respective partners of this complex remain to be confirmed.
